# The Intergenic Interplay between *Aldose 1-Epimerase-Like Protein* and *Pectin Methylesterase* in Abiotic and Biotic Stress Control

**DOI:** 10.3389/fpls.2017.01646

**Published:** 2017-09-25

**Authors:** Ekaterina V. Sheshukova, Tatiana V. Komarova, Denis V. Pozdyshev, Natalia M. Ershova, Anastasia V. Shindyapina, Vadim N. Tashlitsky, Eugene V. Sheval, Yuri L. Dorokhov

**Affiliations:** ^1^Vavilov Institute of General Genetics (RAS) Moscow, Russia; ^2^A.N. Belozersky Institute of Physico-Chemical Biology, Lomonosov Moscow State University Moscow, Russia; ^3^Faculty of Chemistry, Lomonosov Moscow State University Moscow, Russia

**Keywords:** aldose 1-epimerase-like protein, pectin methylesterase, abiotic and biotic stress, virus, bacteria, gene, expression, resistance

## Abstract

The mechanical damage that often precedes the penetration of a leaf by a pathogen promotes the activation of pectin methylesterase (PME); the activation of PME leads to the emission of methanol, resulting in a “priming” effect on intact leaves, which is accompanied by an increased sensitivity to *Tobacco mosaic virus* (TMV) and resistance to bacteria. In this study, we revealed that mRNA levels of the methanol-inducible gene encoding *Nicotiana benthamiana* aldose 1-epimerase-like protein (NbAELP) in the leaves of intact plants are very low compared with roots. However, stress and pathogen attack increased the accumulation of the *NbAELP* mRNA in the leaves. Using transiently transformed plants, we obtained data to support the mechanism underlying AELP/PME-related negative feedback The insertion of the *NbAELP* promoter sequence (proNbAELP) into the *N. benthamiana* genome resulted in the co-suppression of the natural *NbAELP* gene expression, accompanied by a reduction in the *NbAELP* mRNA content and increased PME synthesis. Knockdown of *NbAELP* resulted in high activity of PME in the cell wall and a decrease in the leaf glucose level, creating unfavorable conditions for *Agrobacterium tumefaciens* reproduction in injected leaves. Our results showed that NbAELP is capable of binding the TMV movement protein (MP_TMV_) *in vitro* and is likely to affect the cellular nucleocytoplasmic transport, which may explain the sensitivity of *NbAELP* knockdown plants to TMV. Although NbAELP was primarily detected in the cell wall, the influence of this protein on cellular *PME* mRNA levels might be associated with reduced transcriptional activity of the *PME* gene in the nucleus. To confirm this hypothesis, we isolated the *N. tabacum PME* gene promoter (proNtPME) and showed the inhibition of proNtPME-directed *GFP* and *GUS* expression in leaves when co-agroinjected with the NbAELP-encoding plasmid. We hypothesized that plant wounding and/or pathogen attack lead to PME activation and increased methanol emission, followed by increased *NbAELP* expression, which results in reversion of *PME* mRNA level and methanol emission to levels found in the intact plant.

## Introduction

As sessile organisms, plants are constantly under the pressure of external abiotic (drought, salinity, heat, cold, chilling, freezing, lack of nutrients, high-intensity light) and biotic (microorganisms, insects and herbivores) environmental factors. However, the stress factors for the above-ground and underground parts of the plant and their gene expression profiles are not the same. Wind, rain or herbivorous insects cause damage mainly to stems and leaves, opening the way for penetration of various pathogens. Plant roots suffer primarily from root-feeding herbivores and a lack of water and nutrients. Protective reactions of plants to abiotic and biotic (insects microorganisms and herbivores) environmental factors and their combination are well documented. Unlike the controlled laboratory environment, plants are concurrently affected by more than one abiotic and/or biotic factor in the field, which is an important aspect for evaluating plant stress responses (Suzuki et al., 2014; Ramegowda and Senthil-Kumar, 2015).

In the plant, the intercellular movement of transcription factors and other mobile signals, such as RNA and polypeptides, is important for its normal development (Tilsner et al., 2016). The intercellular movement of macromolecules (RNA and proteins) in the plant is carried out via plasmodesmata (PD), intercellular structures that establish symplastic routes of communication between the neighboring cells in the plant (Tilsner et al., 2016; Brunkard and Zambryski, 2017). Plants, in turn, have created defense mechanisms based on controlling the PD size exclusion limit, which can block the reproduction of viruses (Zavaliev et al., 2013), fungi (Caillaud et al., 2014) and bacteria (Xu et al., 2017). At the same time, the PD is an organelle that participates in the transport of sucrose, which is a product of photosynthesis and plant fixation of carbon dioxide. It is known that the spread of the viral infection and the transport of sucrose are competing events since they use common structures, such as PDs, and common protein factors (Burch-Smith and Zambryski, 2012). One such factor may be aldose 1-epimerase (mutarotase) protein (AEP), the key enzyme of carbohydrate metabolism, which catalyzes the interconversion of alpha- and beta-anomers of sugars such as glucose and galactose (Heese-Peck and Raikhel, 1998). The involvement of an AEP homolog, gp40, in the *Tobacco mosaic virus* (TMV) infection (Lee et al., 2003) and nucleus function (Heese-Peck and Raikhel, 1998) suggests that AEP is a protein, which could be involved in the regulation of host response and intercellular transport.

The cell wall (CW) plays an important role in plant growth and mechanical stress resistance and comprises a dynamic network of polysaccharides and glycoproteins, which determine cell shape, facilitate cell–cell interactions, and provide mechanical strength to plant cells (Corwin and Kliebenstein, 2017). CW pectin forms a matrix around the cellulose–xyloglucan network that is made of rhamnogalacturonan I, rhamnogalacturonan II and homogalacturonan (HG), a major pectic polymer consisting of α-1,4-linked galacturonic acids. HG is secreted in a highly methyl-esterified form and selectively de-methyl-esterified by pectin methylesterases (PMEs) during cell growth and pathogen attack (Micheli, 2001; Pelloux et al., 2007; Wolf et al., 2009a; Lionetti et al., 2012; Lionetti, 2015), resulting in the formation of methanol (Oikawa et al., 2011; Dixit et al., 2013; Dorokhov et al., 2014, 2015; Komarova et al., 2014a). In addition to the ubiquity, the PME is astonishingly abundant in isoforms. For example, *Arabidopsis thaliana* possesses 66 isoforms of PME (Pelloux et al., 2007; Wang et al., 2013; Li et al., 2016). The *PME* gene encodes a PME precursor containing a variable length N-terminal extension that is essential for protein targeting to the endoplasmic reticulum (Markovic et al., 2002). PME maturation requires the removal of the PME leader sequence, including both the transmembrane domain and the spacer sequence (Dorokhov et al., 2006; Wolf et al., 2009b; Mareck et al., 2012). The spacer sequence may either function in subcellular targeting by acting as an intramolecular chaperone for the folding of mature enzymes or as an autoinhibitor during transport through the endomembrane system (Pelloux et al., 2007; Komarova et al., 2014b). The homogalacturonan de-methyl-esterification, catalyzed by PME, is likely to be a feature of plant cell growth control as it leads to constant remodeling and modification after the deposition of the homogalacturonans (Pelloux et al., 2007; Wolf et al., 2009a; Wolf and Greiner, 2012).

The important roles of PME and pectin methyl-esterification in the resistance of plants to insects (Dixit et al., 2013), fungi (Lionetti et al., 2017) and bacteria (Bethke et al., 2014) have previously been demonstrated. The role of PME in viral infection is determined through its interactions with the TMV movement protein (MP) (Dorokhov et al., 1999; Chen et al., 2000), suggesting that PME might be involved in the intercellular movement of TMV via mechanism of piggyback transport of MP through the ER secretory pathway(Chen and Citovsky, 2003).

Mechanical damage to plants dramatically increases the accumulation of *PME* mRNA, and therefore, methanol emission (von Dahl et al., 2006; Körner et al., 2009; Dorokhov et al., 2012). Increased methanol emissions from *PME*-transgenic or mechanically wounded non-transgenic plants were found to retard the growth of the bacterial pathogen *Ralstonia solanacearum* in the neighboring “receiver” plants. Conversely, the overexpression of *PME* in transgenic plants leads to increased levels of methanol in the plant tissue (Dorokhov et al., 2012), which is accompanied by dwarfism in tobacco (Hasunuma et al., 2004).

Methanol has been implicated as a signaling molecule during intraplant and interplant communication by influencing the intact leaves within the same plant and those of the neighboring plants (Komarova et al., 2014b).

The PME activity is likely to be strictly regulated because the state of a permanent cell mobilization depletes the cell's biosynthetic resources and causes generalized changes in the plant. The system must be returned to the state before wounding and pathogen attack. Hypothetically, the regulation of PME occurs at several levels: through the synthesis and stability of the transcript, translation, posttranslational modification and at the level of PME enzymatic activity. The control of the PME enzymatic activity is likely to be performed by the pectin methylesterase inhibitor (PMEI) which forms a specific and stable stoichiometric 1:1 complex with PME *in vitro* (Di Matteo et al., 2005) and *in vivo* (Reca et al., 2012). The interaction of PME and PMEI may be a key part of the mechanism that controls the methyl-esterification status of CW pectin and resistance against TMV (Lionetti et al., 2014a,b) and fungi (Lionetti et al., 2017). The synthesis of *PME* mRNA is highly regulated both during plant growth (Komarova et al., 2014a) and in response to mechanical damage (Dorokhov et al., 2012), however, the mechanism of the *PME* gene control remains unclear.

Previously, we identified the mRNA of a *non-cell-autonomous pathway protein* gene in the leaves of *Nicotiana benthamiana* plants treated with methanol vapor at physiological concentrations (Dorokhov et al., 2012). This gene encodes a protein designated here as *N. benthamiana* AEP-like protein (*NbAELP*) because of its high homology to the gene encoding tobacco AEP gp40 (Heese-Peck and Raikhel, 1998) and other AELP. In the present study, we investigated the possible involvement of *NbAELP* in plant growth and the control of defensive reactions to a pathogen attack. The insertion of the *NbAELP* promoter sequence (proNbAELP) into the *N. benthamiana* genome resulted in the co-suppression of the natural *NbAELP* gene, which was accompanied by a decrease in the glucose level in the leaves, a reduction in the *NbAELP* mRNA content, and increases in PME synthesis and methanol emission. We also showed that NbAELP binds to TMV MP *in vitro* and has the potential ability to influence the cellular nucleocytoplasmic traffic, which is important for understanding the mechanisms underlying methanol-mediated leaf sensitivity to TMV and resistance to bacteria. ProNbAELP is sensitive to methanol and biotic stress. Using transient and stably transformed plants, we obtained data in support of a mechanism by which the *NbAELP* mRNA accumulation induces reduction of *PME* mRNA content in the cell, and, conversely, the decreased level of *NbAELP* expression stimulates the synthesis of both *PME* mRNA and the enzymatically active PME.

## Materials and methods

### Plant growth conditions

*N. benthamiana* and *N. tabacum* plants were grown in soil in a controlled environment under a 16 h/8 h day/night cycle.

### Plasmid and vectors

To obtain the plasmid encoding NbAELP fused to the DYKDDDDKDYKDVDDYKDDDDK (3xFLAG) sequence (Ueda et al., 2011), a 3'-fragment of *NbAELP* containing the 3xFLAG encoding sequence was amplified using NbAELP(BglII+) and FL_SalI_r primers and subsequently digested with BglII and SalI. The 3xFLAG encoding sequence was generated after annealing the primers FL2_SalI_r and FL2_SalI_d, resulting in a fragment with overhangs corresponding to SalI and PstI “sticky” ends. These fragments were inserted into 35S-*NbAELP* via BglII/PstI sites to generate the 35S-*NbAELP:3xFLAG* plasmid. To obtain the proNbAELP (1000)*:GUS* plasmid, NheI and SacI sites were introduced at the 5′- and the 3′-ends of proNbAELP, respectively, through PCR with primers “proNbAELP (NheI+)” and “proNbAELP (SacI−).” The proNbAELP (1,000) NheI/SacI fragment and the *GUS*-35S(term) fragment flanked with SacI/EcoRI sites was cloned into pBIN19, previously digested with XbaI/EcoRI, to generate the proNbAELP(1000):*GUS* construct. To obtain the proNbAELP(1500):*GUS* variant, the upstream region of proNbAELP was amplified using the primers “proNbAELP(SbfI+)” and “proNbAELP (HindIII−)” and subsequently inserted with the proNbAELP(1000):*GUS* fragment, flanked with HindIII/EcoRI sites, into pBIN19, previously digested with SbfI/EcoRI. To obtain proNbAELP(500):*GUS*, the 3′-proximal ~500 nt fragment of proNbAELP, digested with SalI/SacI, and the *GUS*-35S(term) fragment, flanked with SacI/EcoRI, were inserted into pBIN19 via SalI/EcoRI sites.

The proNtPME sequence was amplified using the primer pairs “PMEpr (HindIII+)” and “PMEpr (NcoI−)” and subsequently cloned into the pAL-TA vector (Evrogen, Russia). The obtained proNtPME fragment, with flanking 5′-HindIII and 3′-NcoI sites and the fragment containing *GUS*-35S(term), digested with NcoI/EcoRI, was inserted into pCampia1300 (CAMBIA, Australia) via HindIII/EcoRI sites, resulting in the proNtPME*:GUS* construct. For proNtPME*:GFP* construct, the second used fragment was *GFP*-35S(term) flanked with NcoI/EcoRI sites.

A full list of the oligonucleotides used for cloning is presented in Table S1.

### Transcription start site determination using a step-out rapid amplification of cDNA 5′-end (5′-race) approach

The 5′-RACE of *GUS* cDNA from proNbAELP*:GUS* transgenic plants and *PME* cDNA from wild-type *N. tabacum* plants was performed using the Mint RACE cDNA amplification set (Evrogen, Russia) according to manufacturer's instructions. The following gene-specific primers were used: “GUS rev1”; “GUS rev2”; “PME_rev1”; “PME_rev2”; and “PME_rev3” (Table S1).

### Genome walking to isolate the *NbAELP* and *NtPME* promoter regions

Genomic DNA was isolated from plant tissues using the ZR Plant/Seed DNA MiniPrep™ kit (Zymo Research, USA). A GenomeWalker™ Universal Kit (Clontech, Takara) was used for two rounds of “genome walking” according to the manufacturer's instructions. To identify the *NbAELP* promoter region, the first round of walking was performed using the following oligonucleotides: G05_Rev6, G05_Rev7, and G05_Rev8; the second round of walking was performed using the oligonucleotides G05_Rev10, G05_Rev11, and G05_Rev12 (Table S1). The promoter region fragment was amplified using primers G05_Rev7 and G05_prom_Dir2 and subsequently cloned into the pAL-TA plasmid (Evrogen, Russia).

To identify the *NtPME* promoter region, the first round of walking was performed using the following oligonucleotides: PME_rev4, PME_rev5 and PME_rev6; the second round of walking was performed using the oligonucleotides PME_rev7, PME_rev8 and PME_rev9 (Table S1). The promoter region fragment was isolated using primers PME_rev6 and PMEpr_D and subsequently cloned into the pAL-TA plasmid (Evrogen, Russia).

### Generation of proNbAELP:*GUS* transgenic *N. benthamiana* plants

Agrobacterium-mediated transformation of *N. benthamiana* was performed using the conventional leaf disc method (Horsch and Klee, 1986). Tobacco leaf discs were incubated with *A. tumefaciens* containing the binary vector for 24 h at 26°C in darkness. The infected discs were transferred to regeneration medium (MS medium, supplemented with 1 mg/l 6-benzyladenine and 0.1 mg/l α-naphthalene acetic acid) containing 700 mg/l of cefotaxime and 100 mg/l of kanamycin for selection. Kanamycin-resistant shoots were rooted on selective MS medium containing 100 mg/l of kanamycin. Transgenic T_0_ plants were obtained from *N. benthamiana* discs and characterized through PCR and GUS activity analyses.

### Plant infection with TMV

*N. benthamiana* plants were mechanically inoculated with TMV virions (100 μg/ml) in 50 mM sodium phosphate buffer, pH 7.0, in the presence of celite, as described previously (Dorokhov et al., 1981).

### Agroinjection experiments

The *Agrobacterium tumefaciens* strain GV3101 was transformed with individual binary constructs and grown at 28°C in LB medium supplemented with 50 mg/l rifampicin, 25 mg/l gentamycin and 50 mg/l kanamycin. The *Agrobacterium* from an overnight culture were resuspended in 10 mM MES buffer (pH 5.5) supplemented with 10 mM MgSO_4_ and adjusted to a final OD_600_ of 0.1. Agroinjection was performed using nearly fully expanded *N. benthamiana* leaves attached to the intact plant. A bacterial suspension was infiltrated into the leaf tissue using a 2-ml syringe, after which the plants were grown under greenhouse conditions at 24°C and a 16 h/8 h light/dark photoperiod. The fluorescent cells were counted after 2–3 days of storage in a growth chamber at 24°C under a 16 h/8 h light/dark photoperiod. The bacterial growth was measured by macerating five leaf discs of 1 cm^2^ from the inoculated tissue of each sample in 10 mM MgCl_2_, plating the serial dilutions on nutrient agar plates, and counting the colony-forming units (cfu).

### The renatured blot overlay binding assay

The experiments were performed in accordance with the protocol (Ueki and Citovsky, 2011).

### Semi-thin sections of epon-embedded leaf tissue

The leaf fragments were fixed in 0.1 M Sörensen phosphate buffer (pH 7.4) containing 4 % glutaraldehyde (SPI) for 1.5 h at room temperature, postfixed with 1% OsO_4_ (Sigma) for 2 h and embedded in Epon 812 (Fluka). Semi-thin (1–3 μm) transverse sections were cut using an LKB Ultratome III and stained in solution containing 0.5% methylene blue and 0.5% sodium tetraborate at 60°C for 2 min. The preparations were examined using an AxioVert 200M microscope (Carl Zeiss) equipped with an AxioCam MRc digital camera. For brightness and contract correction, and final presentation, all images were transferred into Adobe Photoshop (Adobe Systems).

### Western-blot analysis

For Western blot analysis, the proteins from agroinjected leaves were divided into S30, P30, P1, and CW crude fractions according to Deom et al. (1990) with modifications. Briefly, frozen plant material was ground to a powder in liquid nitrogen followed by addition of 3 volumes of ice-cold modified GB buffer (100 mM Tris, pH 8.0, 0.4 M sucrose, 10 mM KCL, 5 mM MgCl_2_, 10 mM β-mercaptoethanol). The obtained slurry was filtered through a double-layered Miracloth (Millipore/Merck). The material retained on the filter was collected and washed (30–60 min incubation followed by centrifugation at 1,000 × g) 5–8 times with GB-buffer supplemented with 0.1% Triton X-100 (the final wash was performed without Triton X-100) to obtain the CW-enriched fraction. The filtrate was centrifuged at 1,000 × g for 10 min to obtain the P1 (pellet enriched with nuclei) fraction. Supernatant was further divided into S30 (supernatant enriched with soluble proteins) and P30 (pellet enriched with membrane non-soluble proteins) fractions after centrifugation at 30,000 × g for 30 min. Pellets from CW, P1 and P30 fractions were resuspended in one volume of 1xPBS. To obtain the fraction of apoplast proteins the leaf was infiltrated with buffer containing 100 mM Tris pH 7.0, 150 mM NaCl, 20 mM CaCl_2_, 200 mM mannitol, 0.05% Triton X-100. Then the leaf segments were put into the 0.5 ml tube with the perforated bottom which was inserted into 1.5 ml tube. The apoplast proteins fraction was obtained by centrifugation at 5,000 × g for 5 min.

Aliquotes from all fractions were analyzed through SDS-polyacrylamide gel electrophoresis and blotted onto polyvinylidene difluoride membranes (GE Healthcare). For GFP, NbAELP, FLAG or TMV MP detection, the membranes were probed with corresponding antibodies: goat anti-GFP antibodies conjugated with horseradish peroxidase (Rockland Immunochemicals), mouse anti-FLAG monoclonal antibodies (Sigma), mouse polyclonal antibodies against recombinant NbAELP or TMV MP. Anti-mouse antibodies conjugated with horseradish peroxidase (Rockland Immunochemicals) were used as secondary antibodies. The bands were visualized using the chemiluminescence ECL kit (GE Healthcare). The densitometry of the GFP bands on the Western blots was performed using open-access ImageJ software.

### Gel diffusion assay for the quantification of PME activity

The PME activity in plant samples was quantified through a gel diffusion assay according to Dorokhov et al. (2012). Briefly, the tissue samples were flash-frozen in liquid nitrogen and homogenized in 3 volumes of extraction buffer (1 M NaCl, 2.5 mM phenylmethylsulfonyl fluoride, 0.1 M citrate and 0.2 M sodium phosphate, dibasic, pH 7.0). The homogenate was centrifuged at 16,000 g at 4°C, and the protein concentration in the recovered supernatant was determined using the Bio-Rad protein assay kit. Approximately 30 μl of the homogenate was subsequently loaded onto a 2% (w/v) agarose gel containing 0.1% of 90% esterified pectin (Sigma) in a Petri dish. The gels were incubated for 16 h at 28°C, rinsed with water, and stained for 45 min at room temperature with 0.05% (w/v) ruthenium red dye (Sigma), which stains de-esterified pectin. The diameter of each stained zone was measured to the nearest 0.1 mm using calipers. The amount of PME activity (in nkatals) was calculated based on the standard curve of the log-transformed enzyme activity versus the stained zone diameter generated using a commercial-grade orange peel PME (Sigma).

### Methanol analysis

The methanol emitted by proNbAELP*:GUS* transgenic plants was measured in the headspace of hermetically sealed jars as previously described (Dorokhov et al., 2012). The methanol content was determined through gas chromatography. Methanol emissions was expressed as mg of methanol per 1 g fresh leaf weight.

### Methanol treatment of proNbAELP*:GUS* transgenic plants

The experimental procedures included a 6 h-incubation of proNbAELP*:GUS* transgenic plants with methanol vapors (4 mg of methanol per 20-L sealed dessicator), followed by storage under greenhouse conditions after 18 h and qRT-PCR to examine *GUS* mRNA accumulation.

### Determination of GUS activity

GUS activity was determined using a previously described method (Jefferson et al., 1987) and measured in relative light units. GUS activity was normalized to the protein concentration estimated using a Bio-Rad protein assay kit. The mean values (with SE bars) for 3 to 10 independent experiments are shown.

### Measurement of glucose content in leaves

Dried leaf samples (30 mg) were hydrolyzed with 1 ml of 1M hydrochloric acid (100°C for 2.5 h). The resulting solution was centrifuged for 10 min at 14,000 × g. To 0.5 ml of the supernatant was diluted with 1.5 ml of water and loaded to a reversed-phase concentrating cartridge (Diasorb C16), the first 1.8 ml was discarded and the next 0.2 ml was collected. To 20 μl of a mixture of standards at a concentration of 1 g/L of each carbohydrate (external standard) and test solution, 20 μl of an internal standard solution (glucosamine solution with a concentration of 1 g/l) was added and evaporated on a SpeedVac vacuum centrifugal evaporator with heating in a polypropylene tube. To the dried sample, 20 μl of a 0.5 M solution of PMP (1-phenyl-3-methyl-5-pyrazolone) in methanol and 20 μl of 0.3 M KOH was added, shaken thoroughly and incubated at 70°C for 2 h. The sample was neutralized by the addition of 20 μl of 0.3 M hydrochloric acid and the excess of the PMP reagent was extracted twice with 500 μl of benzene. The residue was evaporated on a SpeedVac with heating and dissolved in 500 μl of acetonitrile/water (1:9). The test mixture and analytical samples (injected vol 10 μL) were analyzed by reversed-phase HPLC in a gradient mode on a Luna C18(2) 4.6 × 250 mm (5 μm) column with the mobile phase A—water, B—acetonitrile and D—100 mM potassium hydrogen phosphate in water (pH 9.12) at a flow rate of 1 ml/min, a temperature of 25°C and with UV detection at 260 nm using a gradient chromatograph Agilent 1,100 with photodiode array detector. Gradient program: 50% D (constant), 8–16% B (5 min), 16–16% B (8 min), 16–30% B (4 min). Total analysis time is 20.65 min. Collection and processing of chromatograms was carried out with ChemStation (Agilent) and AutoChrom1200 (ACDlabs) programs.

### Q-PCR analysis of transcript concentrations

Total RNA was extracted from plant tissues using TriReagent (MRC) according to the manufacturer's instructions. The RNA concentration was determined using a Nanodrop ND-1000 spectrophotometer (Isogen Life Sciences). All RNA samples had a 260:280 absorbance ratio between 1.9 and 2.1. The synthesis of the first strand, followed by real-time qPCR, was performed as described in Dorokhov et al. (2012). Briefly, 0.1 mg of random hexamers and 0.1 mg of oligo-dT primer were added to 2 mg of total RNA to obtain cDNA through reverse transcription performed using Superscript II reverse-transcriptase (Invitrogen), according to the manufacturer's protocol. Real-time quantitive PCR was carried out using the iCycler iQ real-time PCR detection system (Bio-Rad). Target genes were detected using sequence-specific primers (Table S2) and Eva Green master mix (Syntol) according to the manufacturer's instructions. Each sample was run in triplicate, and a non-template control was added to each run. A minimum of five biological replicates were performed.

### Statistics

Student's *t*-test was performed using Excel (Microsoft). *P*-values < 0.05 were considered significant.

### Accession numbers

Sequence data from this article can be obtained from the EMBL data library under accession numbers HG937605 (*N. benthamiana AELP* promoter region) and HG937606 (*Nicotiana tabacum PME* promoter region).

## Results

### Amino acid sequence analysis of NbAELP and its localization in *N. benthamiana* subcellular structures

**Figure 1:**
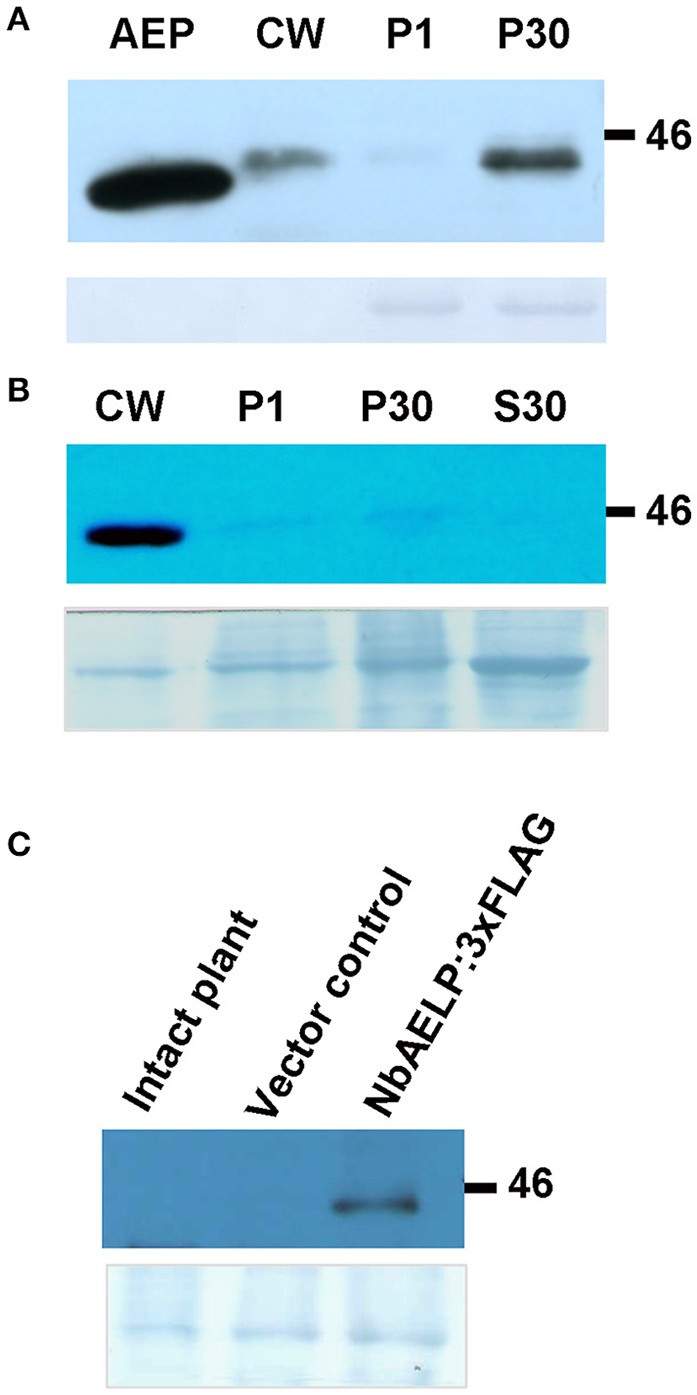
NbAELP of agroinjected leaves was revealed mainly in the cell wall fraction. **(A)** Western blot analysis with NbAELP-specific antibodies of subcellular fractions of *N. benthamiana* leaves at 3 day after agroinjection with *A. tumefaciens* containing “empty” vector (pBin19). AEP, porcine kidney mutarotase “Calzyme” (~400 ng). CW, cell wall fraction; P1, fraction enriched with nuclear proteins; P30, fraction containing mainly membrane proteins from Golgi apparatus and endoplasmic reticulum. **(B,C)** Western blot analysis using anti-FLAG antibodies of *N. benthamiana* leaf proteins at 3 days after agroinjection with the 35S-*NbAELP:3xFLAG* vector. Cell homogenate was fractionated into CW, P1, P30, S30 (fraction of soluble cytoplasmic proteins) **(B)** and apoplast **(C)** fractions. The lower panels show the protein loading control stained with Amido Black.

The analysis of the amino acid sequence of NbAELP classified it in the family of mutarotases (aldose 1-epimerases, EC 5.1.3.3) (Figure S1). High-resolution X-ray structure of the galactose mutarotase from *Lactococcus lactis* indicates the presence of an active site in its structure, which is an open cleft responsible for anchoring the sugar and includes Arg71, His96, His170, Asp243, and Glu304 (Thoden and Holden, 2002; Thoden et al., 2002). All these amino acids are present in the sequence of NbAELP and other AELPs from plants of the *Solanaceae* family. The similarity between NbAELP and the mammalian mutarotase was also confirmed by the ability of the antibodies obtained for the recombinant 6xHis tag containing NbAELP reacted with the porcine kidney mutarotase (Figure 1A). Unlike the mutarotases of bacteria (*L. lactis*) and mammals (porcine kidney), NbAELP contains a signal sequence that is also predicted for AELP of other plants of the *Solanaceae* family (tobacco, potato) (Figure S1). Western blot analysis of the leaf protein reveals NbAELP in membrane-containing P30 and the cell wall (CW) fractions (Figure 1A). However, due to the high homology between different AELPs, antibodies against NbAELP could cross-react with other members of this family, such as the glycoprotein gp40, which is highly abundant in *N. benthamiana* cells, therefore we examined the subcellular localization of NbAELP with a 3xFLAG tag fused to its C-terminus (Figure S2). The 35S*-NbAELP:3xFLAG* construct was introduced into the leaves of *N. benthamiana* plants using agroinjection; after 72 h, the subcellular leaf fractions were analyzed for NbAELP:3xFLAG accumulation. Western blot analysis revealed that the NbAELP:3xFLAG was primarily present in the CW (Figure 1B) and apoplasts (Figure 1C). The NbAELP:3xFLAG was also slightly detected in the P30 membrane and P1 nuclear fractions (Figure 1B). Notably, these results did not preclude the presence of small amounts of NbAELP or its fragments in the nucleus.

### NbAELP mRNA content in the leaves and roots of intact plants

**Figure 2:**
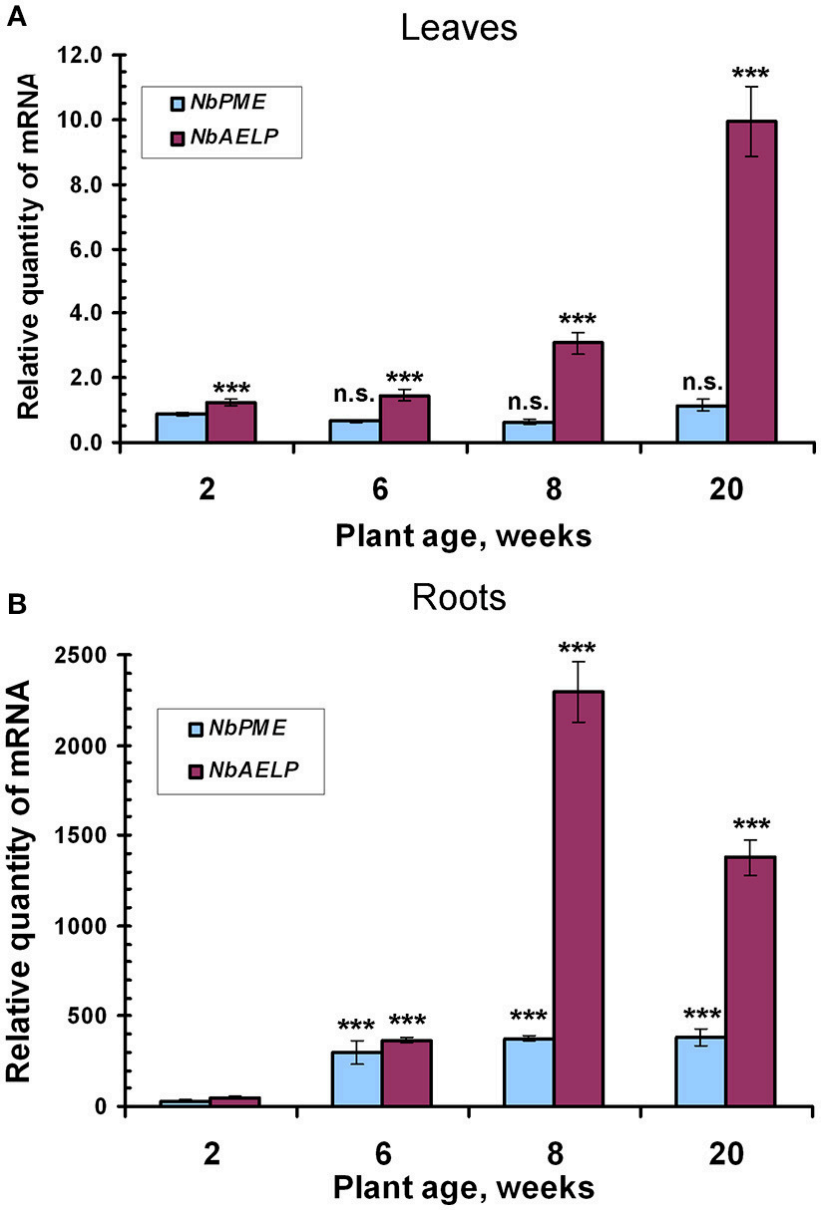
The age-related balance of the *NbPME* and *NbAELP* mRNA content in *N. benthamiana* plants of different ages (Figure S3) starting from seedlings (2 weeks) and ending with flowering (20 weeks) plants. Relative amounts of *NbPME* and *NbAELP* mRNAs in leaves **(A)** and roots **(B)** quantified by qRT-PCR. The data shown represent five independent experiments, with mean values and standard error bars calculated from experimental sets of 10 to 15 plants each. The levels of *NbPME* and *NbAELP* mRNA in leaves normalized to the corresponding mRNAs content in leaves of seedlings. The levels of *NbPME* and *NbAELP* mRNA in roots normalized to the corresponding mRNAs content in leaves from the plant of the same age. Unpaired two-tailed Student's *t*-test *P*-values were used to assess the statistical significance of the difference in *NbAELP* mRNA between 2-weeks seedlings and other plants. ^***^P < 0.001; n.s., not significantly different.

It can be assumed that the synthesis of NbAELP, which affects the functioning of the cell, should be strictly regulated in the intact leaf. We performed an analysis of the NbAELP mRNA in the tissues of the intact *N. benthamiana* plant. Thus, we selected *N. benthamiana* plants of different ages (Figure S3), including 2 (seedling stage), 6, 8, and 20 (flowering plants) weeks, and analyzed the *NbAELP* mRNA content of their leaves and roots. Figure 2 shows the increase in the *NbAELP* mRNA level during the growth of the plant from the seedling to the flowering stage. Although plant growth is accompanied by a modification of the CW, the increase of *NbAELP* mRNA content in leaves (Figure 2A) and roots (Figure 2B) is markedly higher in comparison to PME mRNA. Albeit the *NbAELP* mRNA content in the leaves increases with age, its content in the mature plant is much lower than in the roots.

### *NbAELP* and productive TMV infection

**Figure 3:**
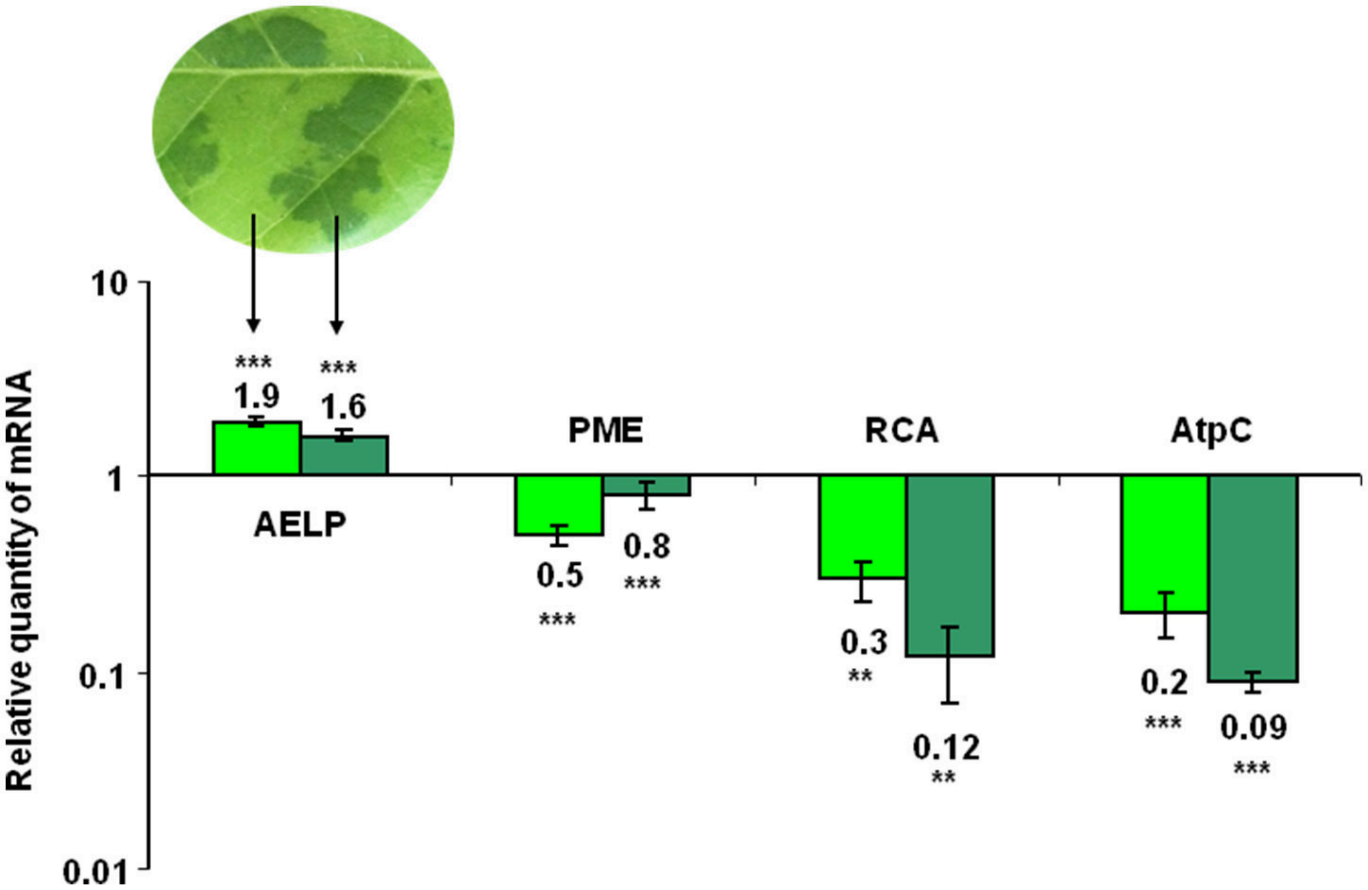
The *AELP* mRNA level in TMV-infected leaves. The qRT-PCR analysis of the *AELP* mRNA levels in the dark green and yellow-green areas of systemically TMV-infected tobacco plants. The diagram shows the log-transformed data of the relative quantity of *AELP, PME, AtpC* (*ATP synthase gamma chain*, chloroplastic), and *RCA* (*Ribulose bisphosphate carboxylase/oxygenase activase*) mRNAs. The levels of analyzed mRNAs of the uninfected *N. tabacum* leaf were used as the controls and set as 1. The data represent five independent experiments, with standard error bars indicated. Unpaired two-tailed Student's *t*-test *P*-values were used to assess the statistical significance of the difference in mRNA between intact and infected leaves. ^***^P < 0.001; ^**^P < 0.01.

Assuming the involvement of NbAELP in viral pathogenesis, we hypothesized that during TMV infection there is a direct correlation between the synthesis of the viral proteins and the accumulation of *NbAELP* mRNA. To verify this assumption, we infected *N. tabacum* plants with TMV and measured the amount of *AELP* mRNA in different parts of the infected leaves. The typical symptom of systemic TMV infection on tobacco is mosaic (Figure S4): dark green islands on the leaves with negligible TMV particles number and the yellow-green areas that are characterized by massive virus accumulation (Moore et al., 2001). Our analysis showed that *AELP* mRNA content increased in infected leaves compared with the intact leaf. Conversely, the content of *PME* mRNA decreased in both the dark green islands and the yellow-green areas of the mosaic leaf. Moreover, we observed the drop in the mRNA levels of the chloroplast genes of the AtpC (*ATP synthase gamma chain*, chloroplastic) and RCA (*Ribulose bisphosphate carboxylase*/*oxygenase activase*) genes in the dark green islands (Figure 3).

We concluded that NbAELP in leaves manifests itself as a pathogenesis-related protein involved in the plant-virus interaction.

### *NbAELP* transcription promoter is sensitive to methanol

**Figure 4:**
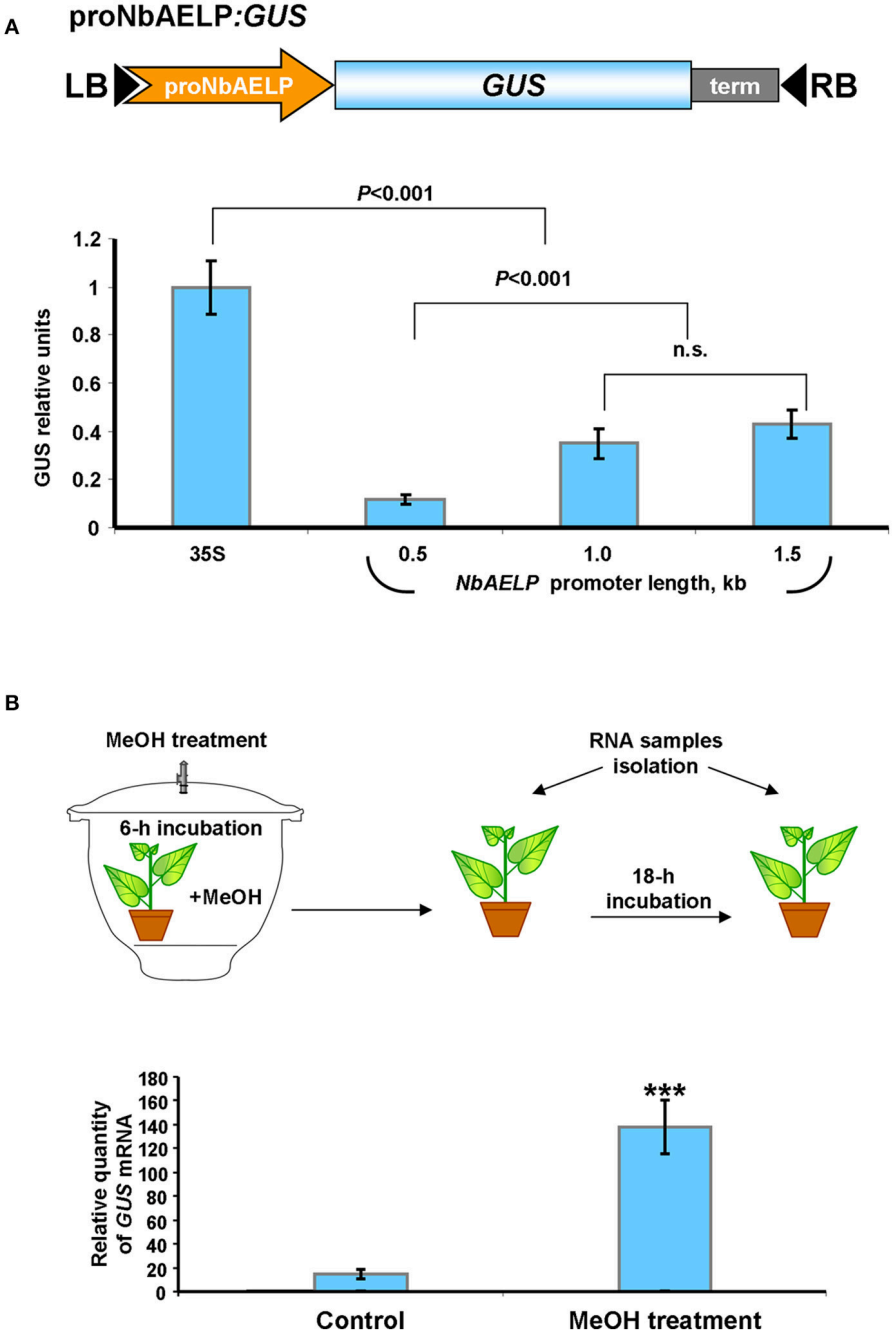
The upstream sequence for *N. benthamiana NbAELP* gene possesses transcriptional promoter activity sensitive to MeOH treatment. **(A)** Schematic representation of a series of proNbAELP-based GUS encoding binary vectors (top) and comparison of the GUS activities in *N. benthamiana* plants agroinjected with these vectors (bottom). GUS activity in plants transfected with proNbAELP-*GUS* binary vectors was compared with the GUS activity in plants transfected with the 35S-*GUS* vector taken for 1.0. The mean values (with SE bars) for five independent experiments are shown. The *P*-values are indicated; n.s., non-significant. LB and RB, left and right T-DNA borders; term, the 35S terminator of transcription. **(B)** MeOH-sensitive synthesis of *GUS* mRNA in the leaves of NbAELPpro-*GUS* transgenic *N. benthamiana* plants. Top: Schematic drawing of the experimental procedures, including the 6-h incubation of proNbAELP*-GUS* transgenic plants with MeOH vapors (4 mg of MeOH) in a sealed 20L desiccator. Control plants were incubated for 6 h in a sealed desiccator without MeOH. Bottom: The *GUS* mRNA content in the leaves of proNbAELP-*GUS* transgenic *N. benthamiana* plants after MeOH treatment. *GUS* mRNA level before incubation with MeOH was set as 1. The data represent five independent experiments, and the standard error bars are indicated. The *P*-values were used to assess the statistical significance of differences in the *GUS* mRNA levels in MeOH-treated plants compared with untreated control plants. ^***^P < 0.001.

Previously, we showed that injury induced *PME* mRNA synthesis leads to the formation of methanol, which in turn stimulates the accumulation of *NbAELP* mRNA (Dorokhov et al., 2012). This suggests that the NbAELP gene promoter is probably sensitive to methanol. To check this hypothesis, we isolated the *N. benthamiana* chromosomal DNA portion with a length of 1,563 base pairs upstream of the *NbAELP* start codon (proNbAELP) and performed a bioinformatics analysis of the sequence. We predicted potential *cis*-regulatory elements that control the gene stress response (Figure S5). In the second step, we created a series of proNbAELP-based binary vectors with varying lengths of the proNbAELP region followed by the *GUS* sequence (Figure 4A, top). The results showed that although proNbAELP directs GUS synthesis, it is inferior to the *CaMV* 35S promoter, which is considered a strong constitutive promoter for achieving high levels of gene expression in dicot plants. The levels of GUS increased with the length of proNbAELP sequence (Figure 4A, bottom).

To assess whether methanol activates proNbAELP, we created transgenic *N. benthamiana* plants expressing *GUS* under the control of proNbAELP. The transgenic plants were incubated in a methanol-rich atmosphere for 6 h and were subsequently maintained under greenhouse conditions (Figure 4B, top). Following this, the accumulation of *GUS* mRNA was analyzed using qRT-PCR. As shown in Figure 4B (bottom), the treatment with methanol dramatically increased the level of *GUS* mRNA.

We concluded that proNbAELP is sensitive to gaseous methanol and can mediate the increased synthesis of *GUS* mRNA in the leaves of transgenic proNbAELP-*GUS* plants in response to methanol.

### Properties of the proNbAELP-*GUS* transgenic *N. benthamiana* plants

The analysis of proNbAELP-*GUS* transgenic *N. benthamiana* plants revealed an extremely low content of endogenous *NbAELP* mRNA (Figure S6). The reduced *NbAELP* mRNA levels likely reflect *NbAELP* gene silencing induced by the transgenic proNbAELP DNA sequence and/or the 5′ non-translated *NbAELP* mRNA sequence, which is located upstream of the *GUS* open reading frame. The analysis of the transcription start site (TSS) localization revealed a 24-nt region of homology between the 5′ non-translated regions of *NbAELP* and proNbAELP*-GUS* mRNAs (Figure S7).

**Figure 5:**
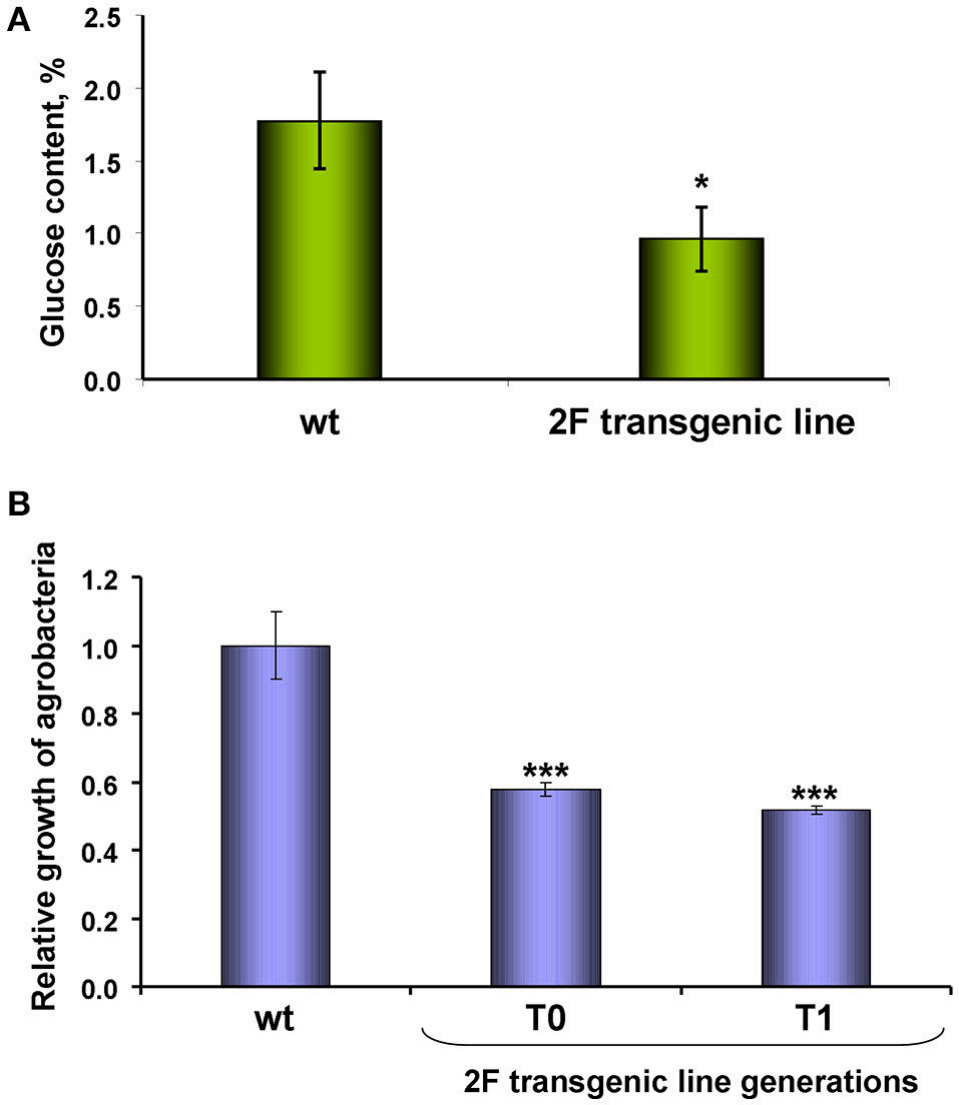
*NbAELP* gene knockdown leads to a decrease in glucose in the leaves and the manifestation of resistance to bacterial infection. **(A)** The content (%) of glucose in the dry leaf samples. The mean values and standard error are shown. **(B)** The relative growth of *A. tumefaciens* in leaves of proNbAELP-*GUS N. benthamiana* transgenic plants, generations T0 and T1, compared with wild-type (WT) control plants on the 3rd day after inoculation. Unpaired two-tailed Student's *t*-test *P*-values were used to assess the statistical significance of the difference between WT and transgenic plants. ^***^P < 0.001; ^*^P < 0.05.

Gene knockdown reveals its function and influence on the biosynthetic processes in the plant. The virus spreading is closely related to the transport of photoassimilates because the PD capacity increase leads to the outflow of sugars from the source leaves to the sink leaves. To assess the potential effect of NbAELP on sugar metabolism, we tested the glucose content in the leaves of the proNbAELP-*GUS* transgenic *N. benthamiana* plants compared with the wild-type. The glucose content of the transgenic plants with reduced *NbAELP* mRNA content appeared to be significantly lower (Figure 5A) than control, indicating the role of *NbAELP* in regulating carbohydrate metabolism.

Biologically, a decrease in glucose levels creates unfavorable conditions for the propagation of the bacterial pathogen (Yamada et al., 2016). To test this assumption, we introduced a suspension of *A. tumefaciens* into the leaf and tested the reproduction of these bacteria. Figure 5B shows that the viability of bacteria introduced into the transgenic leaf almost two times lower.

Thus, in proNbAELP-*GUS* transgenic *N. benthamiana* plants, low glucose in the leaves is correlated to the resistance to *A. tumefaciens* bacteria.

### NbAELP is potentially capable of interacting with TMV MP, but its knockdown leads to increased TMV sensitivity

**Figure 6:**
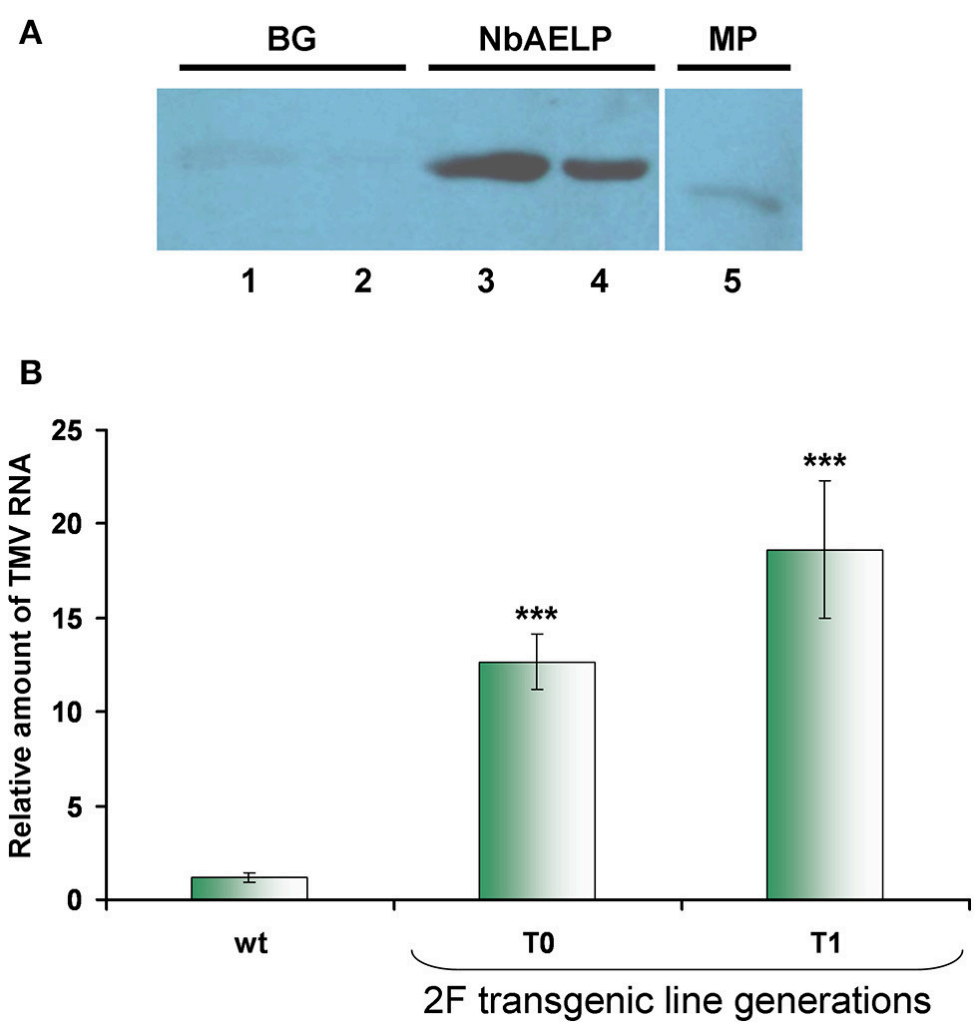
NbAELP/MP binding *in vitro* does not explain the increased sensitivity to TMV of transgenes with a *NbAELP* gene knockdown. **(A)** NbAELP interacts with the TMV MP *in vitro* in the renatured blot overlay binding assay. The TMV MP was tested for binding to proteins immobilized on the membrane: BG, β-1,3-glucanase (lane 1—1.0 μg, lane 2—0.33 μg); NbAELP (lane 3—1.5 μg, lane 4—0.5 μg); TMV MP (lane 5—0.6 μg). Detection was performed with antibodies against TMV MP. **(B)** TMV mRNA accumulation in the virus inoculated leaves of proNbAELP-*GUS N. benthamiana* transgenic plants at the 4th day after inoculation with TMV compared to WT control plants. qRT-PCR analysis of the TMV mRNA content in the leaves of the proNbAELP-*GUS N. benthamiana* transgenic line 2F of T0 and T1 generations. Error bars represent SE calculated from 3 experimental sets of 10 to 15 plants each. The *P*-values were used to assess the statistical significance of the differences compared with WT control plants. ^***^P < 0.001.

Reproduction of the virus depends on the efficacy of viral RNA replication and its intercellular movement. Previously, we showed that methanol enhances the reproduction of TMV, which is associated with the ability of β-1,3-glucanase and NbAELP to stimulate PD gating (Dorokhov et al., 2012). It can be assumed that the direct interaction of TMV MP with β-1,3-glucanase and NbAELP plays an important role in these effects. To verify this assumption, we used the renatured blot overlay binding assay. Figure 6A shows that *in vitro* TMV MP interacts with NbAELP, but does not interact with β-1,3-glucanase, suggesting that differential mechanisms govern the involvement of NbAELP in TMV pathogenesis.

The properties of NbAELP to bind TMV MP and promote PD gating suggest its involvement in TMV reproduction as an MP carrier (Lee et al., 2003). Previously, we showed that NbAELP might increase TMV-directed GFP accumulation due to viral reproduction (Dorokhov et al., 2012). It could be assumed that plants with *NbAELP* knockdown would also be resistant to both TMV and bacteria. However, contrary to our expectations, proNbAELP-*GUS* transgenic *N. benthamiana* plants were highly sensitive to the virus. Figure 6B shows that inoculation with the TMV suspension of the transgenic leaf resulted in a much greater accumulation of viral RNA compared with the leaves of wild-type plants.

The enhanced ability of plants with *NbAELP* knockdown to allow TMV reproduction suggests that NbAELP has an additional ability to influence TMV infection.

**Figure 7:**
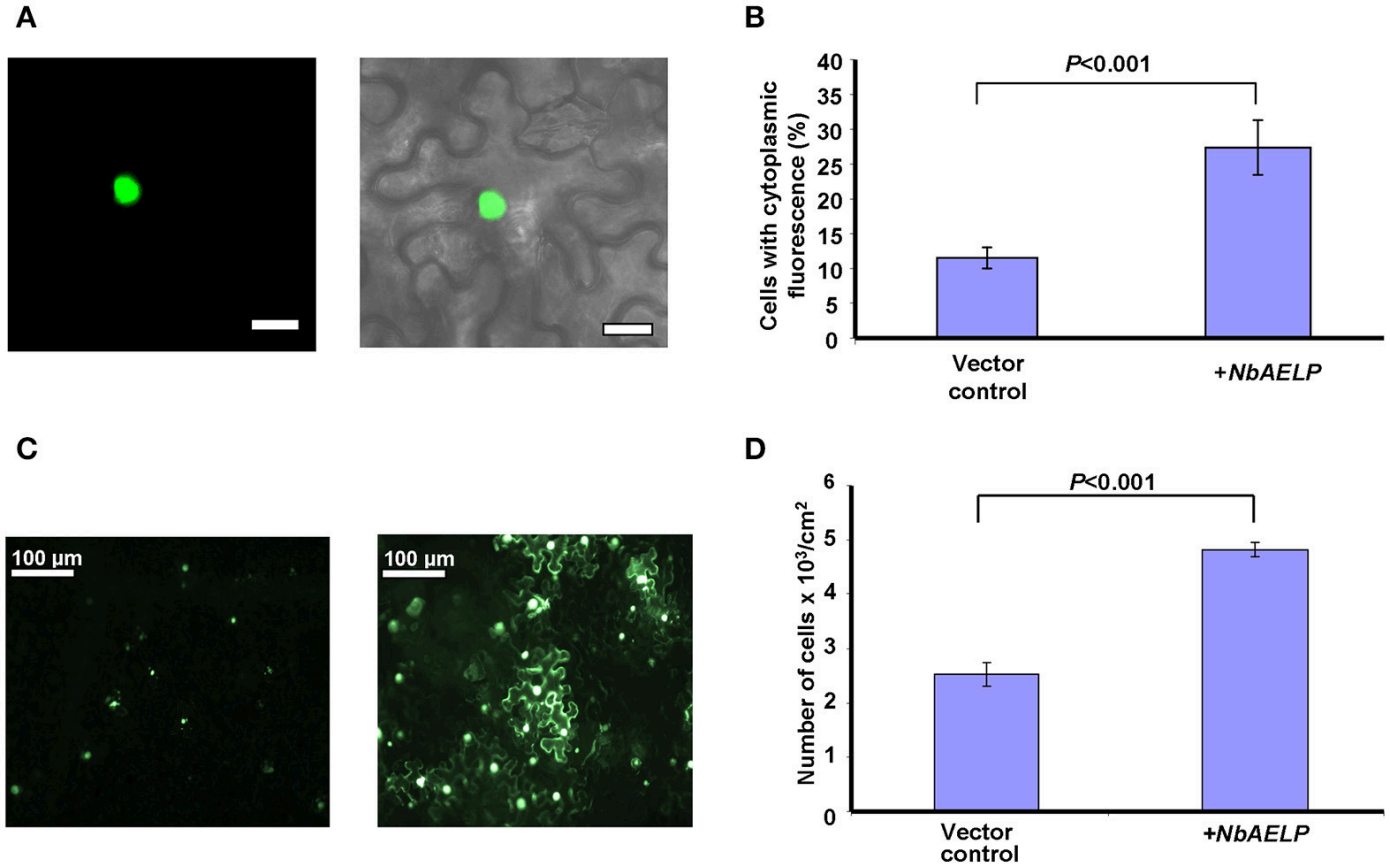
NbAELP is capable of influencing nucleocytoplasmic traffic. **(A)** Visualization of GFP expression in epidermal cells of leaves co-agroinjected with GFP:NLS^pTα^ (left) with a projection of several confocal sections superimposed on a bright field image of the same cell (right). Bars = 20 μm. **(B)** Quantification of GFP:NLS^pTα^-directed GFP subcellular localization in the plant leaves after co-agroinjection with 35S-*NbAELP* or vector control. **(C)** GFP fluorescent epidermal cell cluster under epifluorescence microscopy at 24 h after agroinjection with 35S-GFP:NLS^pTα^ alone (left) or in combination with 35S-*NbAELP* (right). **(D)** Quantification of the cell clusters shown in **(C)**. At least 500 cell clusters were counted for each experiment. The data represent five independent experiments. The standard error bars and *P*-value for the statistical significance of the difference between the vector control and 35S-*NbAELP*-injected leaves are indicated.

We suggested that like tobacco gp40, NbAELP participates in nucleocytoplasmic transport (Heese-Peck and Raikhel, 1998). To determine whether NbAELP functions in the import of nuclear proteins, we used an experimental approach based on the import of GFP fused to the NLS from a nuclear animal protein prothymosin alpha (NLS^pTα^). Figures 7A,B show that *NbAELP* agroinjection prevented the nuclear trafficking of GFP:NLS^pTα^. The presence of GFP in the cytoplasm was not associated with the degradation of GFP:NLS^pTα^ or the appearance of NLS-lacking GFP. Western blot analysis showed the high stability of GFP:NLS^pTα^ fusion proteins (data not shown).

It can be assumed that the NbAELP-induced disruption of nucleocytoplasmic transport could lead to a decrease in plant cell resistance to the foreign nucleic acid and could increase the efficiency of leaf cells transfection. Figure 7C shows that NbAELP increased the number of GFP:NLS^pTα^-containing cells as expected. The quantification of the observed cells with GFP fluorescence revealed a two-fold increase in number of GFP–containing cells per cm^2^ compared with the control (Figure 7D). We conclude that the overexpression of NbAELP can disrupt the nucleocytoplasmic traffic and suppress cell resistance against foreign nucleic acids for example silencing.

### *NbAELP* gene knockdown followed by *NbPME* overexpression leads to dwarfism and leaf structure modifications

**Figure 8:**
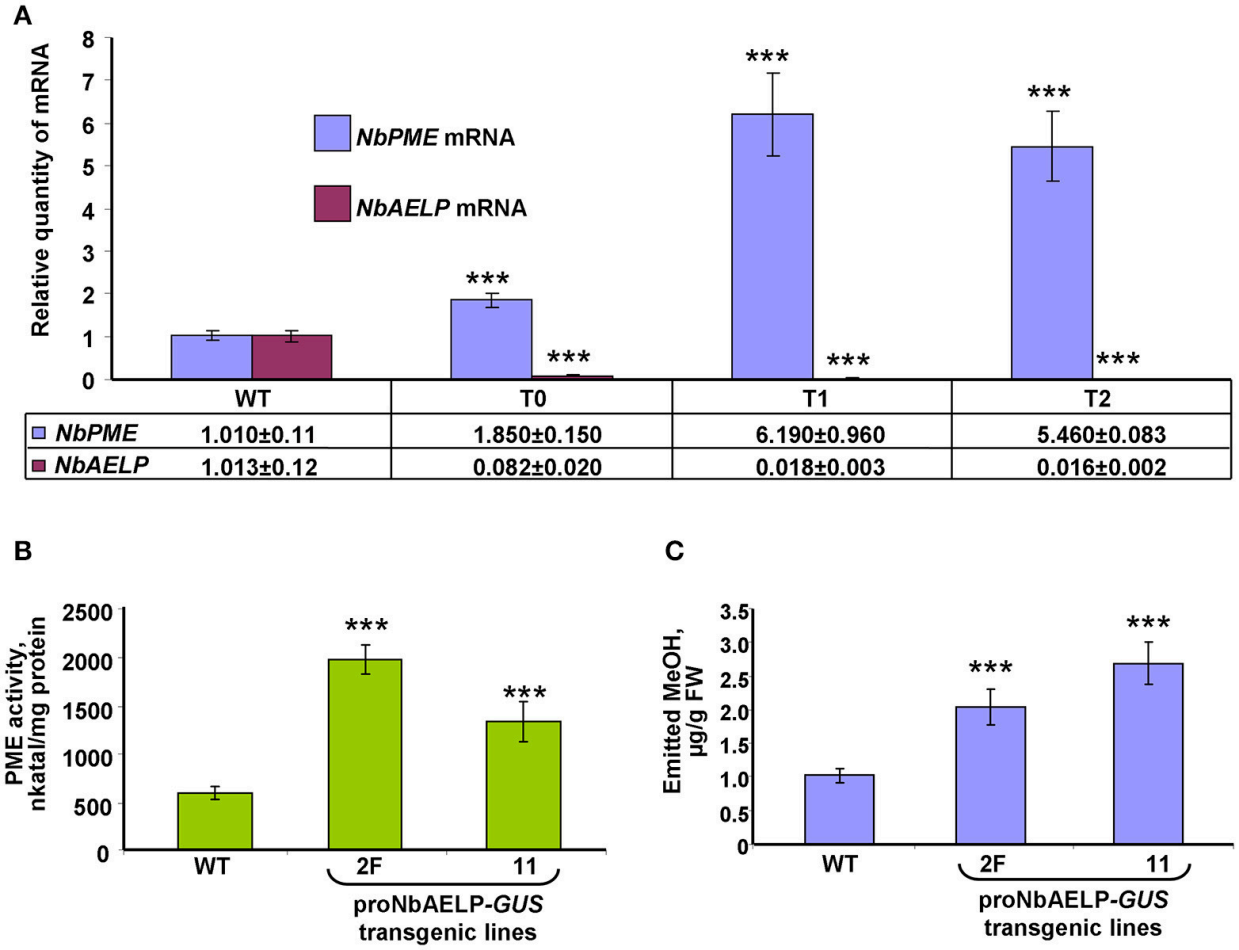
*NbAELP* and *NbPME* mRNA content in the leaves of proNbAELP-*GUS N. benthamiana* transgenic lines compared with WT control plants. **(A)** *NbAELP* and *NbPME* mRNA content in the leaves of the proNbAELP*-GUS N. benthamiana* transgenic line 2F of generations T0, T1, and T2. The means of the corresponding mRNAs levels of the WT plants were set as 1. **(B,C)** PME enzymatic activity **(D)** and MeOH emission **(E)** of proNbAELP-*GUS N. benthamiana* transgenic lines 2F and 11. All data represent the average values of five independent measurements, and the standard error bars are indicated. The *P*-values were used to assess the statistical significance of the differences compared with WT control plants. ^***^ *P* < 0.001.

**Figure 9:**
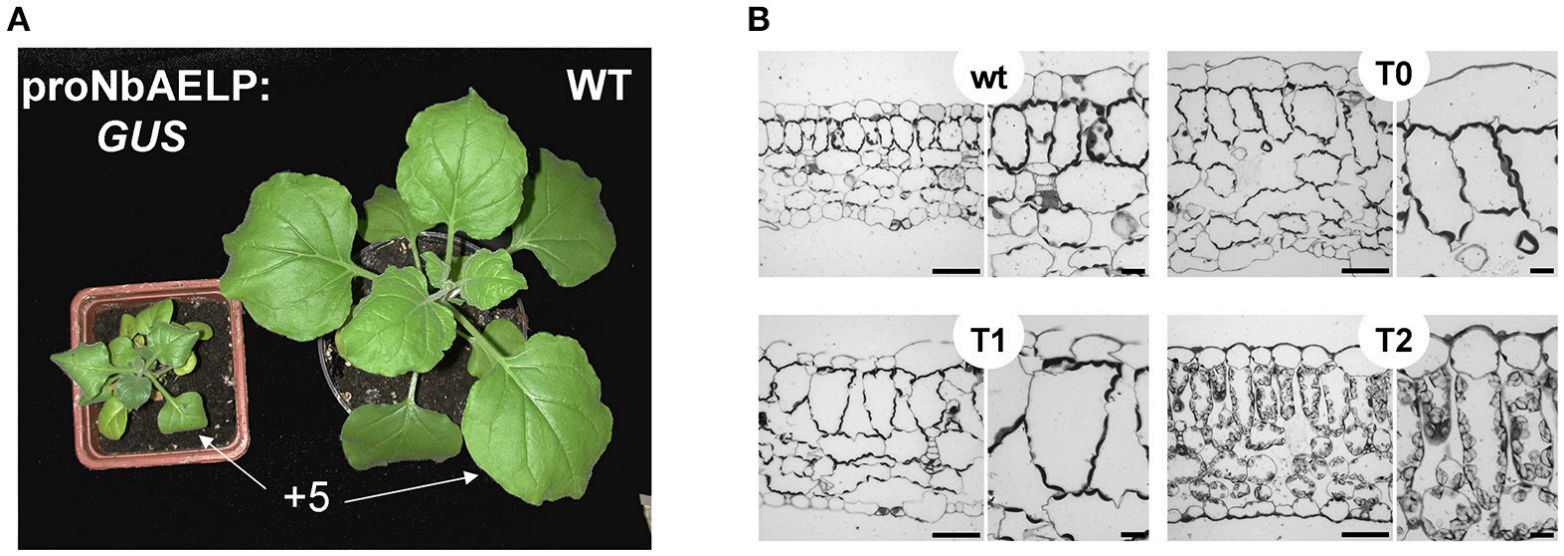
The proNbAELP-*GUS* transgenic *N. benthamiana* plants manifest dwarfism and leaf structure alteration. **(A)** Aerial phenotype of the proNbAELP*-GUS* transgenic *N. benthamiana* 2F line of generation T1 at 5 weeks after sowing compared with WT control plants. The location of +5 leaf used for area measurement (Table 1) is marked. **(B)** Transverse section of Epon-embedded leaf tissue from the proNbAELP-*GUS* transgenic *N. benthamiana* 2F line of generations T0, T1 and T2; bar = 50 μm.

To understand the role of NbAELP in a viral infection, we explored the close, competitive relationship between NbAELP and NbPME. Our results showed that *NbAELP* mRNA decrease in the proNbAELP-*GUS* transgenic *N. benthamiana* plants was related to the increase in *NbPME* mRNA (Figure 8A), the high level of PME enzymatic activity in the CW (Figure 8B) and the increase in methanol emission (Figure 8C). The long-term presence of high PME levels in CWs can result in dwarfism in plants (Hasunuma et al., 2004). In the present study, the proNbAELP*-GUS* transgenic *N. benthamiana* plants showed signs of dwarfism (Figure 9A, Table 1) like those observed with 35S*-PME* transgenic *N. tabacum* plants (Figure S8A). Moreover, the high CW PME content leads to an increase in the size of cells in 35S*-NtPME* transgenic tobacco leaves (Figure S8B) and proNbAELP-*GUS* transgenic *N. benthamiana* leaves (Figure 9B, Table 1). In both types of transgenic plants, the leaves and the cuticle were thicker and showed significant starch deposition compared with the wild-type.

**Table 1:** Quantification^a^ of the plant height, leaf and cell sizes of transgenic proNbAELP-*GUS* plants (T2) compared to WT *N. benthamiana*.

***N. benthamiana* plant**	**Height, cm**	**Leaf area^b^, cm^2^**	**Cell length^c^, μm**
WT	11.40 ± 0.79	12.21 ± 0.76	34.92 ± 1.38
proNbAELP-*GUS*	5.27 ± 0.39^#^	3.94 ± 0.45^#^	72.41 ± 2.19^#^

a *The data shown represent mean values and standard errors calculated from at least 10 plants, 20 cells per plant*.

b *The area of +5 leaves (Figure 9A) from plants of the same age was measured*.

c *The length of palisade parenchyma cells*.

# *P < 0.001, unpaired two-tailed Student's t-test was used to assess the statistical significance of the difference between transgenic proNbAELP-GUS plants (T2) and WT N. benthamiana*.

### *NbAELP* agroinjection of *N. benthamiana* plants suppresses endogenous *NbPME* mRNA accumulation in the leaves

**Figure 10:**
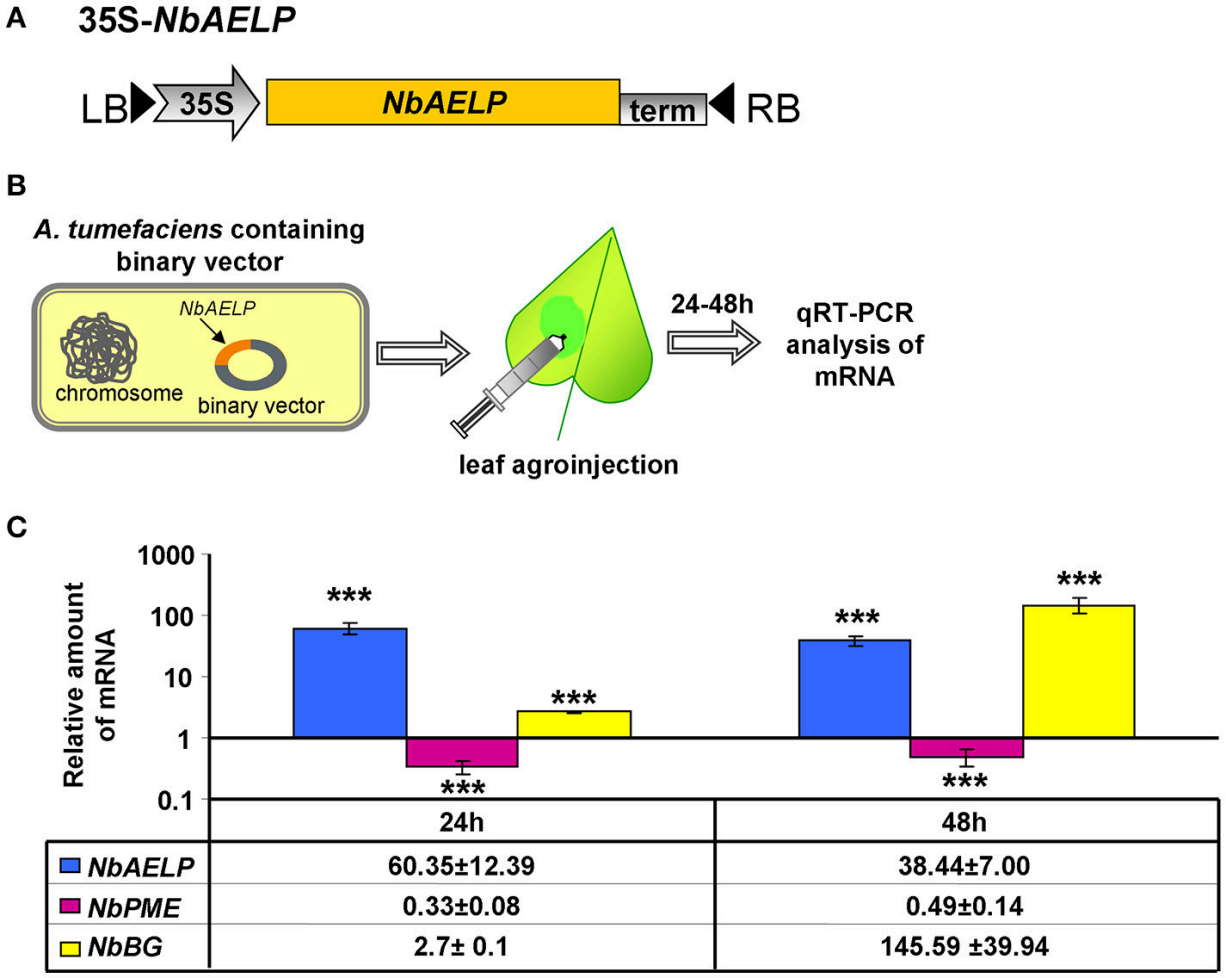
*N. benthamiana* leaf agroinjection with *NbAELP* suppresses endogenous *NbPME* mRNA accumulation. **(A)** Schematic representation of the *NbAELP* plasmid (35S-*NbAELP)*. **(B)** Agroinjection experiments (from left to right): *A. tumefaciens* containing a binary vector with the *NbAELP* gene under 35S promoter control; leaf agroinjection using a needleless syringe containing agrobacterium suspension from an overnight culture; subsequent mRNA isolation and qRT-PCR analysis. **(C)** qRT-PCR analysis of the mRNA levels in the leaves of *N. benthamiana* plants agroinjected with 35S-*NbAELP*. The semi-log plot shows the measurements of the relative quantities of *NbAELP* and endogenous *NbPME* and β*-1,3-glucanase* (*NbBG*) mRNA, obtained using qPCR. The mean of the “empty vector” control was set to 1. Error bars represent SE calculated from 3 experimental sets of 10 to 15 leaves each. The *P*-values were used to assess the statistical significance of the differences in the mRNA levels compared with “empty vector” control. ^***^P < 0.001.

Biologically, if there is a relationship between *NbAELP* and *NbPME* at the mRNA level, a methanol-induced increase in *NbAELP* mRNA synthesis should also cause a change in the accumulation of *NbPME* mRNA within the cells. To directly verify this assumption, *N. benthamiana* plants were agroinjected with an *NbAELP*-encoding binary vector (Figure 10A), and the endogenous level of *NbPME* mRNA was measured using qRT-PCR (Figure 10B). Figure 10C shows the accumulation of *NbAELP* mRNA and the consequential reduction of endogenous *NbPME* mRNA in the leaves of 35S-*NbAELP* agroinjected plants. An unpaired two-tailed Student *t*-test was used to confirm that there was a statistically significant difference in the *NbPME* mRNA levels in leaves taken from control plants versus 35S-*NbAELP*-agroinjected plants. This effect cannot be explained by a non-specific degradation of mRNA because the endogenous mRNA levels of the β*-1,3-glucanase* gene are sensitive to cellular stress (Dorokhov et al., 2012) and do not increase following agroinjection (Figure 10C).

Thus, the observed relationship between *NbAELP* and *NbPME* mRNA levels in proNbAELP-GUS transgenic plants was confirmed by experiments when *NbAELP* gene overexpression suppressed the accumulation of *NbPME* mRNA in the cytoplasm.

### NbAELP downregulates mRNA accumulation directed by *PME* transcription promoter

To unravel the relationship between NbAELP and PME, we hypothesized a mechanism in which NbAELP influences the *NbPME* mRNA synthesis. To determine whether AELP influences *PME* gene transcription, we isolated *N. tabacum* chromosomal DNA nucleotide sequence upstream of the *NtPME* gene (proNtPME) and located the TSS (Figure S9A). To confirm that the 1.75-kb long DNA region that is upstream of the *NtPME* gene promotes mRNA synthesis, we constructed a binary vector encoding GUS (Figure S9B) and then measured GUS activity in the leaves of *N. benthamiana* plants agroinjected with proNtPME*-GUS*. Figure S9C shows that proNtPME can mediate GUS synthesis, although the activity of this promoter was 5 times lower than that of the strong constitutive *CaMV* 35S promoter. Thus, we concluded that proNtPME possesses promoter transcriptional activity.

**Figure 11:**
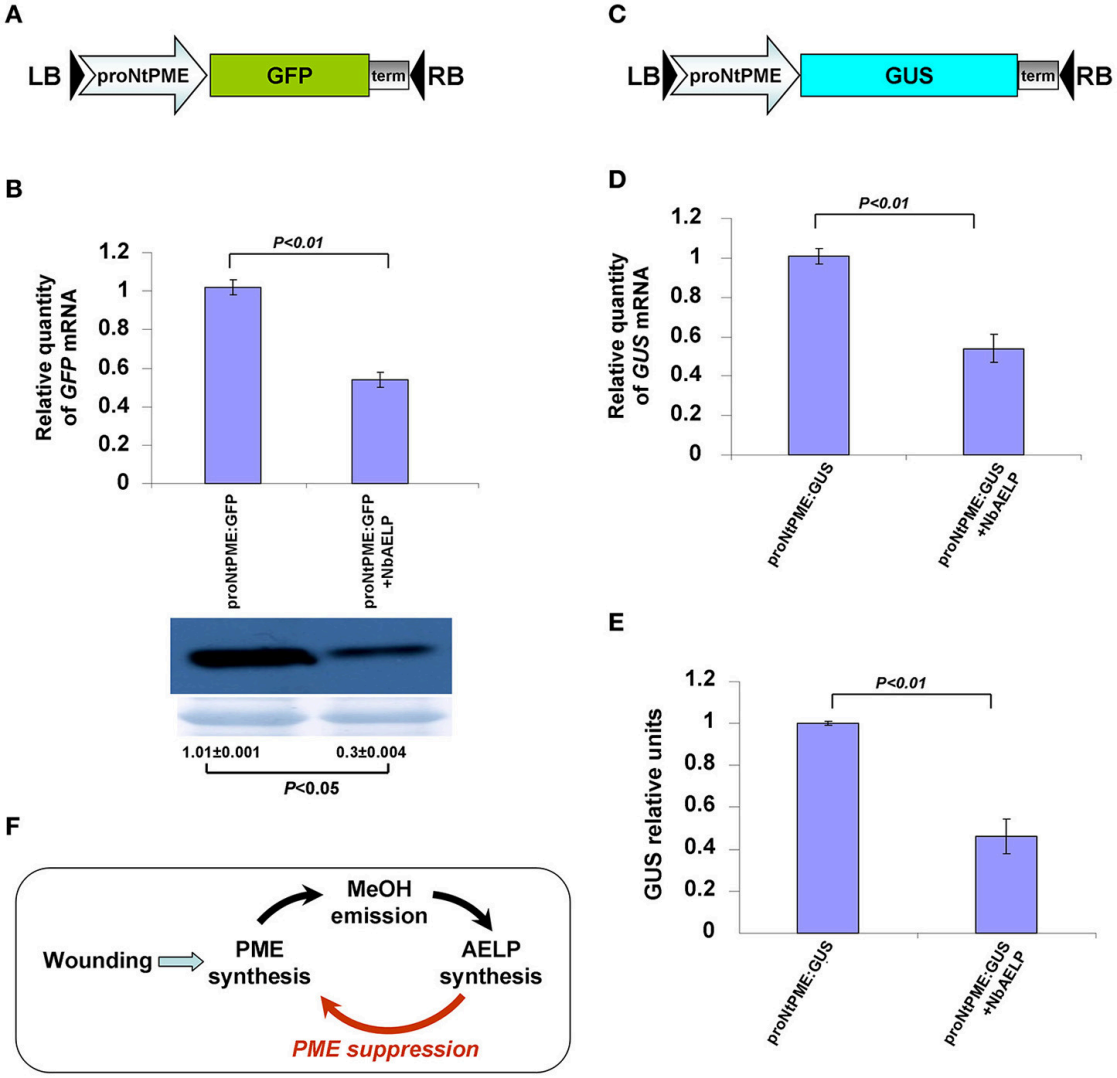
*NbAELP* inhibits the accumulation of proNtPME-directed mRNAs. **(A)** Schematic representation of proNbAELP-based GFP encoding binary vector. LB and RB indicate the left and right T-DNA borders, respectively; 35S, 35S *CaMV* promoter; term, the 35S terminator of transcription. **(B)** *GFP* mRNA content revealed through qRT-PCR (top), and Western blot analysis of the total soluble proteins using anti-GFP antibodies (bottom) in *N. benthamiana* leaves at 3 days after simultaneous agroinjection of proNtPME-*GFP* and 35S-*NbAELP*. The results of densitometry analysis, in relative units, and the standard errors are indicated for the Western blot. The data represent five independent experiments. The *P*-values were used to assess the statistical significance of the differences in the GFP bands and the *GFP* mRNA levels. **(C)** Schematic representation of proNbAELP-based *GUS* gene encoding binary vector. **(D,E)** *GUS* mRNA content revealed through qRT-PCR **(D)**, and the GUS activity measured in relative light units **(E)** in *N. benthamiana* leaves at 3 days after simultaneous agroinjection of proNtPME-*GUS* and 35S-*NbAELP*. The *P*-values were used to assess the statistical significance of the differences in the *GUS* mRNA levels or GUS activity; n.s., non-significant. **(F)** Schematic representation of the PME-AELP relationship in the *Nicotiana* plants.

To support the hypothesis that NbAELP may modify the transcriptional activity of proNtPME, we co-injected plants with a binary vector encoding NbAELP and a proNtPME*-GFP* vector, which had *GFP* under the control of proNtPME (Figure 11A). Agroinjecting plants with proNtPME-*GFP* alone did not result in visible leaf fluorescence, but *GFP* expression could be detected through Western blot analysis. The GFP bands were analyzed using densitometry to estimate the amount of the synthesized GFP. Figure 11B shows that NbAELP inhibited the accumulation of both *GFP* mRNA and the corresponding protein. Notably, NbAELP did not affect the accumulation of 35S-directed *GFP* mRNA (data not shown).

In the next series of experiments, we used a construct that had *GUS* in place of the *GFP* gene (Figure 11C). *N. benthamiana* leaves that were simultaneously agroinjected with proNtPME-*GUS*, and 35S-*NbAELP* showed decreased proNtPME-driven *GUS* expression at the mRNA (Figure 11D) and protein (Figure 11E) levels. The control experiments demonstrated no influence of NbAELP on GUS synthesis driven by the 35S promoter (Figure S10).

Thus, we concluded that NbAELP could suppress proNtPME-driven mRNA accumulation, and therefore, downregulates the synthesis of *PME* mRNA by the feedback principle as shown in Figure 11F.

## Discussion

PME participates in remodeling the plant cell wall during plant growth and stress responses (Pelloux et al., 2007; Wolf et al., 2009a; Wolf and Greiner, 2012). Long and continuous exposure to stress factors usually causes irreversible changes in the plant. For example, a chronic wind accompanied by thigmomorphogenesis and mechanical perturbation of plants leads to modifications of woody plants (Hamant and Moulia, 2016). Short-term stresses also lead to changes in the cells, but these changes are transient, not causing the depletion of cells biosynthetic resources and generalized changes in the plant (Bostock et al., 2014).

Biologically, after short-term stress, the cell is returned to its initial state when the enzymatic activity of PME, with the participation of methanol, returns to the state found in the intact leaf. PMEI plays an important role in controlling PME activity. Recently, this role was most clearly illustrated when studying the infection of *A. thaliana* by the necrotrophic pathogen *Botrytis cinerea* (Lionetti et al., 2017). A feedback loop was suggested in which methanol down-regulates the expression of AtPMEIs to favor the rapid and efficient induction of PME-mediated defense reactions.

Here, our work allows us to propose an additional mechanism for regulating PME activity that is associated with the role of a methanol-inducible gene involved in intercellular transport. We have previously shown that an effective innate immune strategy used by plants against pathogen attack involves intercellular communication, PME synthesis and methanol emission (Dorokhov et al., 2012). This strategy resulted in the accumulation of mRNAs from methanol-inducible genes, including mRNA from the *non-cell-autonomous pathway protein* gene (Dorokhov et al., 2012) that we designated as NbAELP because of high homology with representatives of the mutarotase family (Figure S1).

In contrast to bacterial (Fukasawa et al., 2012) and mammalian (Park et al., 2007) AEPs, the structure and properties of mutarotases of higher plants are not known. NbAELP is probably involved in the metabolism of sugars as suggested by the decreased glucose levels in the leaves of transgenic plants with a decreased level of *NbAELP* mRNA (Figure 5A). NbAELP contains the amino acid signs of mutarotases and common antigenic determinants, similar to the AEP of bacteria and mammals (Figure 1A), but has a signal sequence unlike the other AEPs (Figure S1). The prediction of N-glycosylation sites of NbAELP and its high homology to the glycoprotein gp40 characterizes it as a secreted glycoprotein that passes through the Golgi apparatus during the maturation stages and eventually localizes in the cell wall (Figure 1).

Like AELP, many other cross-kingdom proteins acquire new functions during evolution and expand their functional repertoire; moreover, they may lose their basic function, but acquire a new function (Linkeviciute et al., 2015), as corroborated by examples from virology (Koonin et al., 2015).

NbAELP acquired the ability to influence PD gating probably by participating in the carbohydrate metabolism of the plant (Figure 5A), as evidenced by (i) its involvement in the control of cell-to-cell trafficking (Dorokhov et al., 2012) and (ii) binding of TMV MP *in vitro* (Figure 6A). The function of NbAELP in intercellular transport cannot be ruled out as during post-translational modification and acquisition of N-glycans it indirectly interacts with the PD resident, β-1,6-N-acetylglucosaminyl transferase (Zalepa-King and Citovsky, 2013), which catalyzes the attachment of the oligosaccharide side chains to glycoproteins (Nagels et al., 2011, 2012). Additionally, *NbAELP* probably expanded its functional repertoire and acquired the ability to influence plant immunity (like PR proteins) by acting on nucleocytoplasmic traffic of macromolecules involved in host defense response (Figure 7). *NbAELP*, which is otherwise inactive in the intact leaves (Figure 2), is activated after trauma or pathogen attack (Figure 3). The participation of NbAELP in abiotic and biotic stress responses also implies a negative regulation of the synthesis of PME and methanol (Figure 11E). The inhibition (Figure 8) and an increase of NbAELP mRNA accumulation (Figure 10) affect PME and respectively stimulate or suppress its expression, demonstrating the negative correlation between these two genes. Our experiments with the proNtPME-based constructs have shown that NbAELP can suppress the synthesis of *GFP* and *GUS* mRNA directed by the *NtPME* promoter (Figure 11).

The mechanism of the effect of NbAELP on the expression of the *PME* gene is not clear. Although the NbAELP homolog, gp40, was detected in the nucleus (Heese-Peck and Raikhel, 1998), we were unable to detect an appreciable amount of NbAELP in the nuclei-enriched fraction (P1) (Figure 1).

Biologically, the negative relationship between PME and NbAELP can manifest not only at the level of mRNA synthesis but also at the stage of intracellular traffic, since both proteins probably use common mechanisms of glycosylation and secretion. Our modeling experiment using *N. benthamiana* plants agroinjected with binary vectors encoding NbAELP and a chimeric protein comprising the leader of the PME precursor (Dorokhov et al., 2006) confirmed the ability of NbAELP to compete with PME for its secretion and maturation (data not shown). At the same time, since the leader part of premature PME contains potential glycosylation sites (Figure S11) and the mature PME enzyme processing occurs in the Golgi apparatus, it can compete with NbAELP for the factors of intracellular traffic (Strasser, 2016).

Our proposed model (Komarova et al., 2014b) suggested that the methanol-triggered PD dilation should enhance viral spread within the plant because the methanol-inducible genes, including *NbAELP*, activated cell-to-cell communication, TMV RNA accumulation and resistance to bacteria (Dorokhov et al., 2012). According to this model, TMV reproduction should be suppressed in plants where *NbAELP* expression is inhibited. However, our experiments showed the opposite result (Figure 6B) wherein a decrease in the *NbAELP* mRNA level appeared to be favorable for TMV propagation. This contradiction should be explained at the level of cell-to-cell transport since reproduction of the virus is a result of at least two events, including the replication of the virus in the primary infected cell and the intercellular transport of the viral genetic material. NbAELP function in cell-to-cell communication is likely to be duplicated, though its loss can be compensated by other factors. For example, considering the negative correlation between *PME* and *NbAELP* expression, NbAELP function of the TMV MP piggyback receptor may be performed by PME (Chen et al., 2000), the synthesis of which drastically increases in plants with *NbAELP* knockdown. Thus, we assume that the relationship between NbAELP and PME and their balance is important for maintaining the immune status of a plant.

## Author contributions

YD and TK conceptualized the topic and designed the experiments. EkVS and DP performed most of the experiments. TK, EuVS, VT, NE, and AS conducted some experiments. YD, EkVS, and TK evaluated the data and drafted the skeleton of manuscript. YD, EkVS, and TK revised and finalized the manuscript. All the authors read and approved the manuscript.

### Conflict of interest statement

The authors declare that the research was conducted in the absence of any commercial or financial relationships that could be construed as a potential conflict of interest.

## Supplementary material

Figure S1 Sequence alignment of the deduced amino acid sequence of NbAELP (NCBI accession # C7C5S9) with other homologous proteins. Sequences were retrieved from the National Center for Biotechnology Information with the following accession numbers: O48971, NtAEPL_gp40 (83.10% identity); Q947H5, NtNCAPP1 (95.22% identity); A0A1J6ILZ7, NaAELP (95.52% identity); Q947H4, NtNCAPP2 (95.52% identity); M0ZLG2, StAELP (87.64% identity); K4BBT7, SlAELP (86.92% identity); Q9GKX6, porcine kidney Aldose 1-epimerase (38.94% identity); Q9X762, L. lactis Aldose 1-epimerase (25.15% identity). NetNGlyc 1.0 Server (http://www.cbs.dtu.dk/services/NetNGlyc/) predicted N-glycosylated sites are marked with bold red (Asparagines) and blue (Asn-Xaa-Ser/Thr sequons). Letters in bold show amino acid residues responsible for anchoring the sugar to the protein (Thoden and Holden, 2002). The putative signal sequence is underlined.

Figure S2 Schematic representation of the 35S-*NbAELP:3xFLAG* vector.

Figure S3 View of *N. benthamiana* plants, of different ages, isolated from the soil of the pot.

Figure S4 Yellow-green areas of TMV U1-infected tobacco plant exhibiting typical mosaic symptoms contain a significant amount of coat protein (CP). **(A)** A TMV U1-infected tobacco plant exhibiting typical mosaic symptoms at 8 dpi. Close-up view of the area bounded by the circle is shown in a separate panel where yellow-green (1) and dark green (2) areas are shown. **(B)** Coomassie blue staining of 15% SDS–PAGE of proteins from yellow-green (1) and dark green (2) areas of systemically infected leaves shown on **(A)**. TMV CP (1.5 μg) and proteins of healthy leaves were used as positive and negative controls respectively.

Figure S5 Cis-regulatory elements of *NbAELP* gene promoter. *NbAELP* gene 5' upstream sequence (GenBank: HG937605.1) was analyzed with the Nsite tool (Shahmuradov and Solovyev, 2015). Predicted motifs of plant transcription factor binding sites are marked with orange bars. Transcription start site (TSS) was identified experimentally using the 5′RACE method.

Figure S6 *NbAELP* and *NbPME* mRNA content in the leaves of proNbAELP-*GUS N. benthamiana* transgenic lines compared with WT control plants; qRT-PCR analysis of the mRNA levels in the leaves of proNbAELP:*GUS* transgenic lines 2A, 4, 10, 2F, and 11 of generation T1.

Figure S7 5′RACE analysis of mRNA of proNbAELP:*GUS* transgenic plant. Schematic structure of the proNbAELP:*GUS* genomic insertion comprising the following elements: LB and RB, the left and right T-DNA borders, respectively; proNbAELP, 1-kb-proNbAELP; term, the 35S terminator of transcription. The transcription start site (TSS) and GUS coding sequence (cds) are indicated.

Figure S8 Increased PME enzymatic activity in the CW results in *Nicotiana* plant dwarfism. **(A)** Aerial phenotype of the 35S-NtPME transgenic tobacco at 15 weeks after sowing compared with WT control plants. **(B)** Transverse section of Epon-embedded leaf tissue from 35S-*NtPME* transgenic tobacco; bar = 50 μm.

Figure S9 A 1.75-kb *NtPME* gene upstream sequence directs *GUS* mRNA synthesis. **(A)** Schematic representation of the upstream *NtPME* gene sequence (proNtPME) with two transcription start sites designated as TSS-1 and TSS-2. The start of *PME* coding sequence (cds) is indicated. **(B)** Schematic representation of the binary proNtPME-based GUS encoding vector proNtPME-*GUS*. **(C)** Comparison of the GUS activity in *N. benthamiana* leaves infiltrated with proNtPME-GUS or with 35S-*GUS*. The GUS activity, measured as relative light units, was normalized to the amount of plant total soluble protein. The mean values (with SE bars) for five independent experiments are shown.

Figure S10 NtAELP has no effect on 35S-directed GUS synthesis.

Figure S11 Predicted NbPME N-glycosylated sites with NetNGlyc 1.0 Server (http://www.cbs.dtu.dk/services/NetNGlyc/) are marked with bold red (Asparagines) and blue (Asn-Xaa-Ser/Thr sequons).

Table S1 Oligonucleotides used for cloning, 5'RACE and “genome walking.”

Table S2 Oligonucleotides used for qPCR.
