# Supplementary material for: The Intergenic Interplay between Aldose 1-Epimerase-Like Protein and Pectin Methylesterase in Abiotic and Biotic Stress Control
Source: Front Plant Sci. 2017 Sep 25;8:1646. doi: 10.3389/fpls.2017.01646 (PMC5622589; doi:10.3389/fpls.2017.01646)
Supplement: Figure S1 — Sequence alignment of the deduced amino acid sequence of NbAELP (NCBI accession # C7C5S9) with other homologous proteins. Sequences were retrieved from the National Center for Biotechnology Information with the following accession numbers: O48971, NtAEPL_gp40 (83.10% identity); Q947H5, NtNCAPP1 (95.22% identity); A0A1J6ILZ7, NaAELP (95.52% identity); Q947H4, NtNCAPP2 (95.52% identity); M0ZLG2, StAELP (87.64% identity); K4BBT7, SlAELP (86.92% identity); Q9GKX6, porcine kidney Aldose 1-epimerase (38.94% identity); Q9X762, L. lactis Aldose 1-epimerase (25.15% identity). NetNGlyc 1.0 Server (http://www.cbs.dtu.dk/services/NetNGlyc/) predicted N-glycosylated sites are marked with bold red (Asparagines) and blue (Asn-Xaa-Ser/Thr sequons). Letters in bold show amino acid residues responsible for anchoring the sugar to the protein (Thoden and Holden, 2002). The putative signal sequence is underlined. [file Image1.PDF]

```
L.lactis            --------------MEI-TTKDFGLGSSLISLTNKNDVTIAFTNLGARIVD-----WQKD    40
S.scrofa            ---------MVSVTRSVFGDLPSGAGTVEKFQLQSDQLRVDIISWGCTITALEVKDRQGR    51
NtAELP_gp40         MSLKI-NLLVCLFIFHLLVAASVRGHKIGIYEIKKGDFSVKITNYGTSIISVLLPDKHGK    59
NtNCAPP1            MSSKI-NLLICLFILHLCAAASVRGRKIGIYEIKKGDFSVKITNYGASIISVFLPDKHGK    59
NaAELP              MSSKISLLLICLFILHLCAAASVRGRKIGIYEIKKGDFSVKITNYGASIISVFLPDKNGK    60
NbAELP              MSSKIGLLLICLFILHLCAVSSVRGRKIGICEIKKGDFSVKITNYGATIISVFLPDKHGK    60
NtNCAPP2            MSSKISLLLICLFILHLCAAASVRGRKIGIYEIKKGDFSVKITNYGATIISVLLPDKHGK    60
StAELP              MSSKISLLICLFFILHLLAADSVTGHKIEIYEIKKGDFSVKVTNYGATIISVLLPDKNGK    60
SlAELP              ------------MGADQFGRQVFFCSHAGIYEIKKGDFSVKVTNYGATIISVLLPDKNGK    48
                                                     :..:. : . . *  *        :

L.lactis            GKHFILGFDSAQEYLEKDAYPGATVGRTAGRIKDGLVDISGKTYHLNQNEAPQTLHGGED    100
S.scrofa            ASDVVLGFAELKEYLQKHPYFGAVVGRVANRIAKGTFTLDGKEYKLAINNGPNSLHGGVR    111
NtAELP_gp40         IGDVVLGYDTIEEYKNDTSYFGATLGRVANRIGGAQFTLNGIHYKLVPNEGKNMLHGGPK    119
NtNCAPP1            IGDVVLGYDTIKEYKNDTSYFGAALGRVANRIGGAQFTLNGTHYKLVANEGVNMLHGGLK    119
NaAELP              IGDIVLGYDTIKEYKNDTSYFGAALGRVANRIGGAQFTLNGTHYKLVANEGVNMLHGGLK    120
NbAELP              IGDVVLGYDTIKEYKNDTSYFGAALGRVANRIGGAQFTLNGTLFKLVANEGVNMLHGGLK    120
NtNCAPP2            IGDVVLGYDTIKEYKNDTSYFGAALGRVANRIGGAQFTLNGTHYKLVANEGVNMLHGGLK    120
StAELP              IGDVVLGYDTIKEYRNDTSYFGAALGRVANRIGGAQFTLNGTHYKLVANEGVNMLHGGLK    120
SlAELP              IGDVVLGYDTIKEYKNDTSYFGAALGRFANRIGGAQFTLNGTHYKLVANEGVNMLHGGLK    108
                      ..:**:   :** :.  * **.:** *.**  . . :.*  ::*  *:. : ****

L.lactis            SIHTKLWTYEIN-DLGDEVQVKFSLVSNDGENGYPGKIEMSVTHSFDE-ENNWKIKYEA-    157
S.scrofa            GFDKVLWTPRVLS-----NGIEFSRVSPDGEEGYPGELKVWVTYTLDGGEL--VVNYRA-    163
NtAELP_gp40         GFSKVVWKVSKYVKDGPCPYITLTYYSADGEEGFPGAVLASVTYTLKD-SYKLSVVFRAK    178
NtNCAPP1            GFSKVVWKVSKYVRHGPSPYITLTYYSADGEEGFPGDVLVSVTYALK-DPYKLSVVFKAR    178
NaAELP              GFSKVVWKVSKYVRHGPSPYITLTYYSADGEEGFPGDVLVSVTYALKDNPYKLSVVFKAR    180
NbAELP              GFSKVVWKVSKYVRHGPSPYITLTYYNADGEEGFPGDVLASVTYALK-DPYKLSVVFKAR    179
NtNCAPP2            GFSKVVWKVSKYVRHGPSPYITLTYYSADGEEGFPGDVLVSVTYALK-DPYKLSVVFKAR    179
StAELP              GFSKVVWKVRKYVQDGPSPYITLTYHSADGEEGFPGDVVVSVTYALKD-HYNLSVVFKAK    179
SlAELP              GFSKVVWKVSKYVQDGPSPYITLTYHSADGEEGFPGDVVVSVTYALKD-HYKLSVVFKAK    167
                    .: . :*.            : ::  . ***:*:** :   **:::.       : :.*

L.lactis            ISDKDTVFNPTGHVYFNLNGDASKSIENHQLKLAASRFVPLKDQTEIVRGDIVDTKNTDL    217
S.scrofa            QASQTTPVNLTNHSYFNLAGQGSPNIYDHEVTIEADAFLPVDE-TLIPTGEIAPVQGTAF    222
NtAELP_gp40         ALNKATPINLSHHPYWNIGGHDSGDVLSQVLQIFGSHITLVDK-QLIPTGEIAPIKNTPY    237
NtNCAPP1            ALNKATPINLSHHPYWNIGGHNTGDVLSQVLQIYASHITPLDN-QHIPTGEISPVKNTPY    237
NaAELP              ALNKATPINLSHHPYWNIGGHNSGDVLSQVLKIYASHITPLDE-QHIPTGEISPVKNTPY    239
NbAELP              SLNKATPINLSHHPYWNIGGHNSGDVLSQVLQIYASHITPLDK-QHIPTGEISPVKNTPY    238
NtNCAPP2            ALNKATPINLSHHPYWNIGGHNSGDVLSQVLQIYASHITPLDK-QHIPTGEISPVKNTPY    238
StAELP              ALNKATPINLSHHPYWNIGGHNSGDVLSQVVQIFASHITPLDT-QHIPTGIISPVKNTPY    238
SlAELP              ALNKATPINLSHHPYWNIGGHNSGDVLSQVVQIFASHITPLNT-QHIPTGIISPVKNTPY    226
                      .: *  .* : * *:*: *. : .: .: : : .. :  :.    *  * *   :.*

L.lactis            DFRQEKQLSKALESSMEQVQLVGGIDHPFLLDEQSLEKEQARLS--LDDLSVSVYTDQPS    275
S.scrofa            DLRKPVELGKHLQEF-----HINGFDHNFCLKRSKEKQFCARVHHAGSGRVLEVYTTQPG    277
NtAELP_gp40         DFLKPRKVGSRINKL------KNGYDINYVLDSTEKMKPVGIVYDKKSGRVMDVQASSPG    291
NtNCAPP1            DFLKPHKVGSRINKI------QNGYDINYALDSSKKMKPVAIVYDKKSGRVMDIKATAPG    291
NaAELP              DFLKPHKVGSRIDKI------QNGYDINYALDSNKKMKPVAIVYDKKSGRVMNIQATAPG    293
NbAELP              DFLKPHKVGSRIDKI------QNGYDINYALDSSKKMKPVAIVYDKKSGRVMNIQATAPG    292
NtNCAPP2            DFLKPRKVGSRIDKI------QNGYDINYALDSSRKMKPVAIVFDKKSGRVMDIKATAPG    292
StAELP              DFLKPRKVGSRIDKI------QNGYDINYALDSTEKMKHVAIVYDKKSGREMDIKATAPG    292
SlAELP              DFLKPRKVGSRIDKI------QNGYDINYALDSTEKMKHVAIVYDKKSGRVMDIKATAPG    280
                    *: :  ::.. ::.        .* *  : *.     :  . :    ..  :.: :  *.

L.lactis            IVIFTANFGDLGTV-YHGKNQVHHGGITFECQVSPGSQQIPELGDISLKAGDEYQATTIY    334
S.scrofa            IQFYTGNFLDGTLKGKTGAVYPKHSGFCLETQNWPNAVNQPHFPPVLLKPGEEYNHTTWF    337
NtAELP_gp40         VQFYTANFVNN-TKGKGGFVYQPHSALSLETLVFPDAVNHPNFPSTIVNPGEKYVHSVLY    350
NtNCAPP1            VQFYTANFVIN-TKGKGGYVYQPHSALCLETQGFPDAVNHPNFPSTIVTPGKTNVHSVLY    350
NaAELP              VQFYTANFVIN-TKGKGGYVYQPHSALCLETQGFPDAVNHPNFPSTIVTPGKTYLHSVLY    352
NbAELP              VQFYTANFVIN-TKGKGGYVYQPHSALCLETQGFPDAVNHPNFPSTIVTPGKTYLHSVLY    351
NtNCAPP2            VQFYTANFVIN-TKGKGGYVYQPHSALCLETQGFPDAVNHPNFPSTIVIPGKTYLHSVLY    351
StAELP              VQFYTANWVIN-TKGKGGYVYQPHSALCLETQGFPDAVNHPNFPSTIVTPGKSYVHSVLY    351
SlAELP              VQFYTANFVIN-TKGKGGYVYQPHSALCLETQGFPDAVNHPNFPSTIVTPGKSYVHSVLY    339
                    : ::*.*:         *     *..: :*    *.: : *.:    :  *.    :. :

L.lactis            SLHTN--     339
S.scrofa            VFSVA--     342
NtAELP_gp40         TFSIKK-     356
NtNCAPP1            TFSIKKY     357
NaAELP              TFSIKK-     358
NbAELP              TFSIKK-     357
NtNCAPP2            TFSIKK-     357
StAELP              TFTIKKY     358
SlAELP              TFSIKKY     346
                     :
```
