# Supplementary figures and images for: The Intergenic Interplay between Aldose 1-Epimerase-Like Protein and Pectin Methylesterase in Abiotic and Biotic Stress Control
Source: Front Plant Sci. 2017 Sep 25;8:1646. doi: 10.3389/fpls.2017.01646 (PMC5622589; doi:10.3389/fpls.2017.01646)

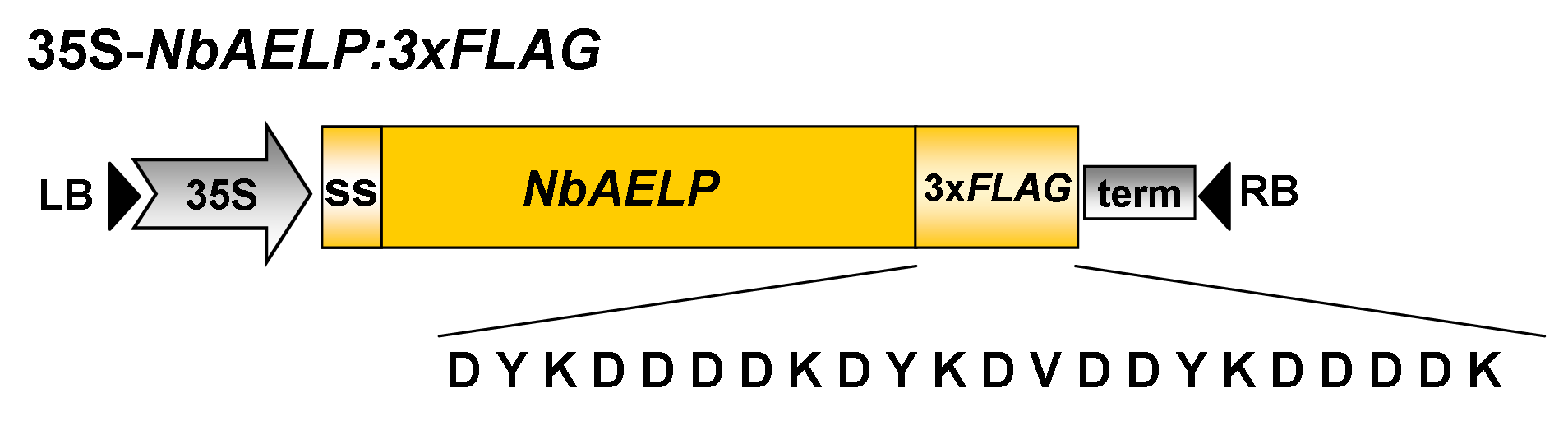

Supplement: Figure S2 — Schematic representation of the 35S-NbAELP:3xFLAG vector. [file Image2.TIF]

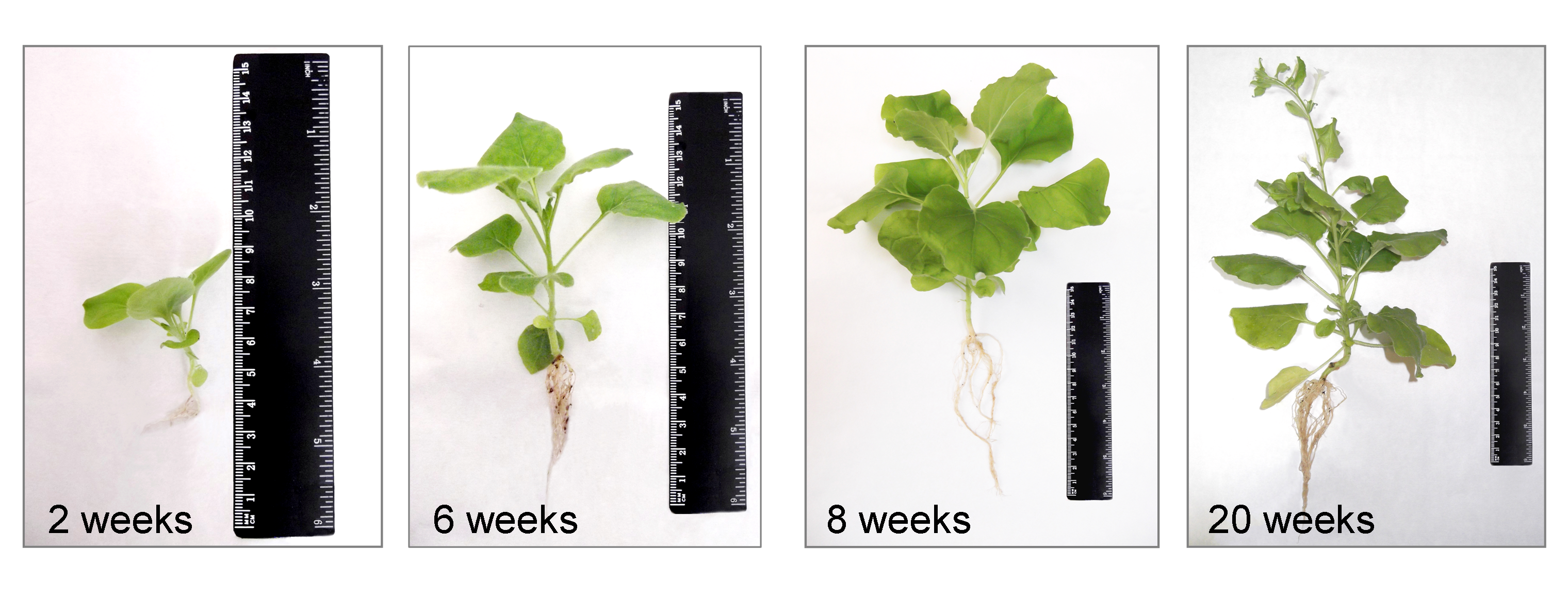

Supplement: Figure S3 — View of N. benthamiana plants, of different ages, isolated from the soil of the pot. [file Image3.TIF]

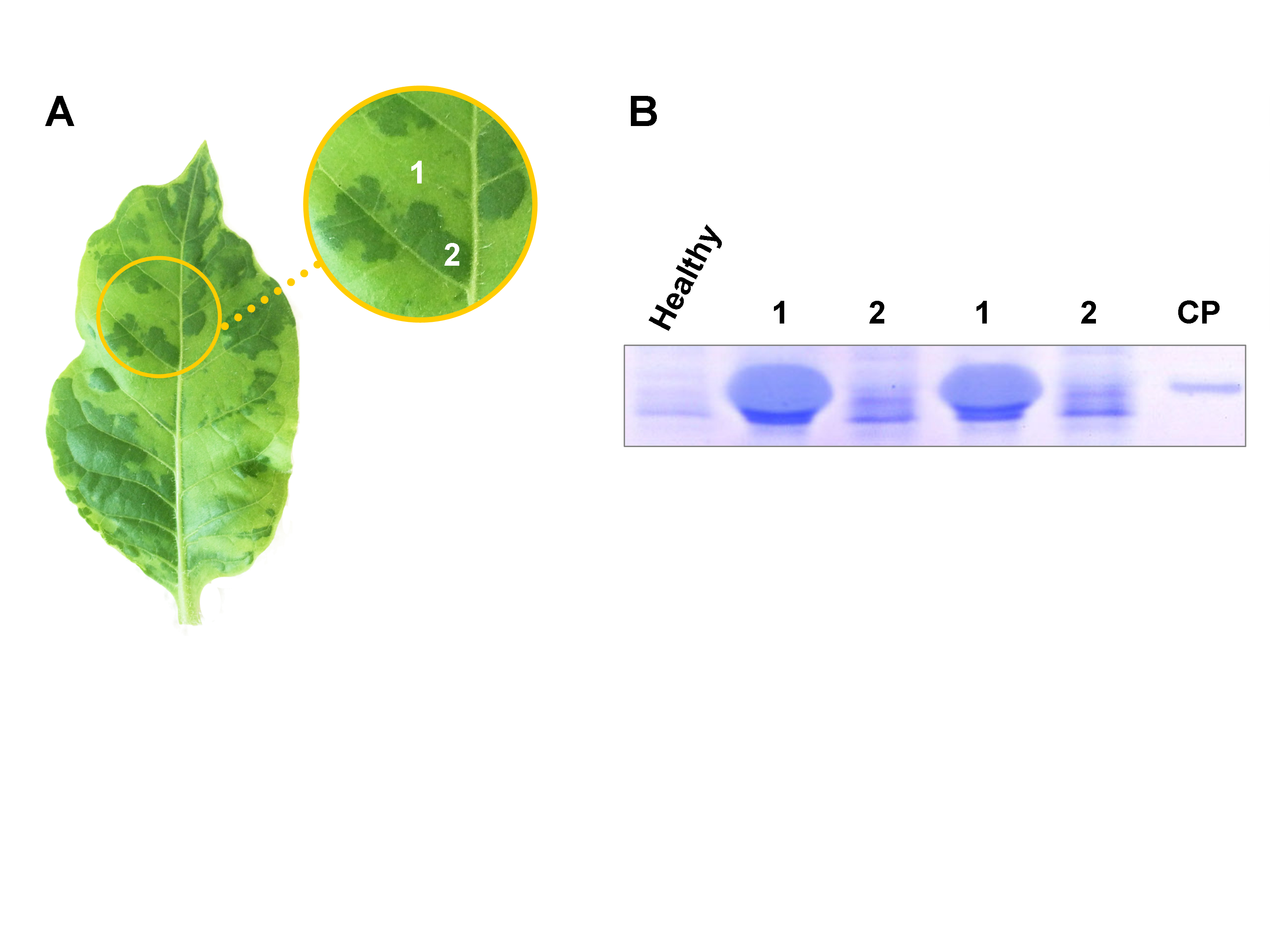

Supplement: Figure S4 — Yellow-green areas of TMV U1-infected tobacco plant exhibiting typical mosaic symptoms contain a significant amount of coat protein (CP). (A) A TMV U1-infected tobacco plant exhibiting typical mosaic symptoms at 8 dpi. Close-up view of the area bounded by the circle is shown in a separate panel where yellow-green (1) and dark green (2) areas are shown. (B) Coomassie blue staining of 15% SDS–PAGE of proteins from yellow-green (1) and dark green (2) areas of systemically infected leaves shown on (A). TMV CP (1.5 μg) and proteins of healthy leaves were used as positive and negative controls respectively. [file Image4.TIF]

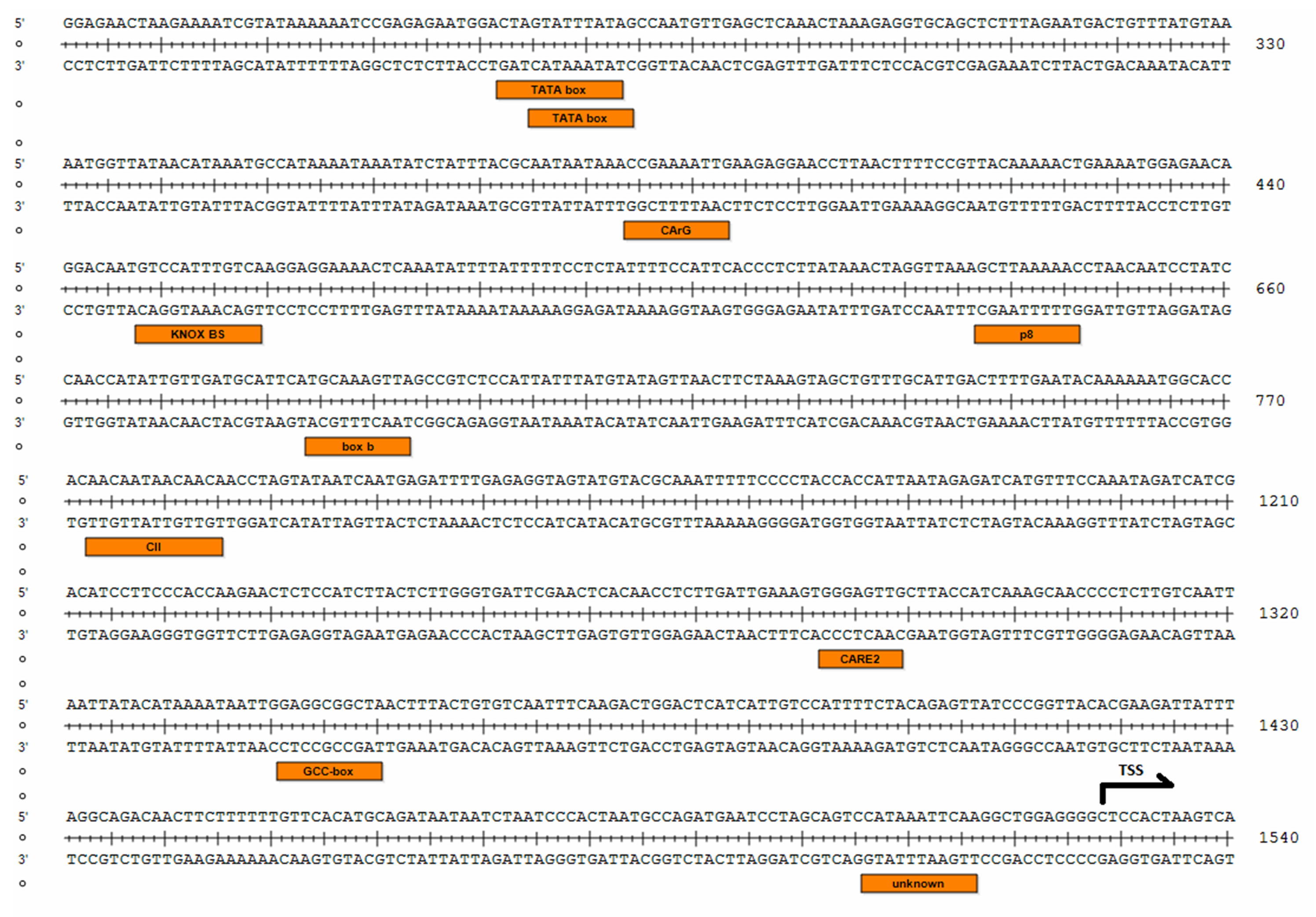

Supplement: Figure S5 — Cis-regulatory elements of NbAELP gene promoter. NbAELP gene 5' upstream sequence (GenBank: HG937605.1) was analyzed with the Nsite tool (Shahmuradov and Solovyev, 2015). Predicted motifs of plant transcription factor binding sites are marked with orange bars. Transcription start site (TSS) was identified experimentally using the 5′RACE method. [file Image5.TIF]

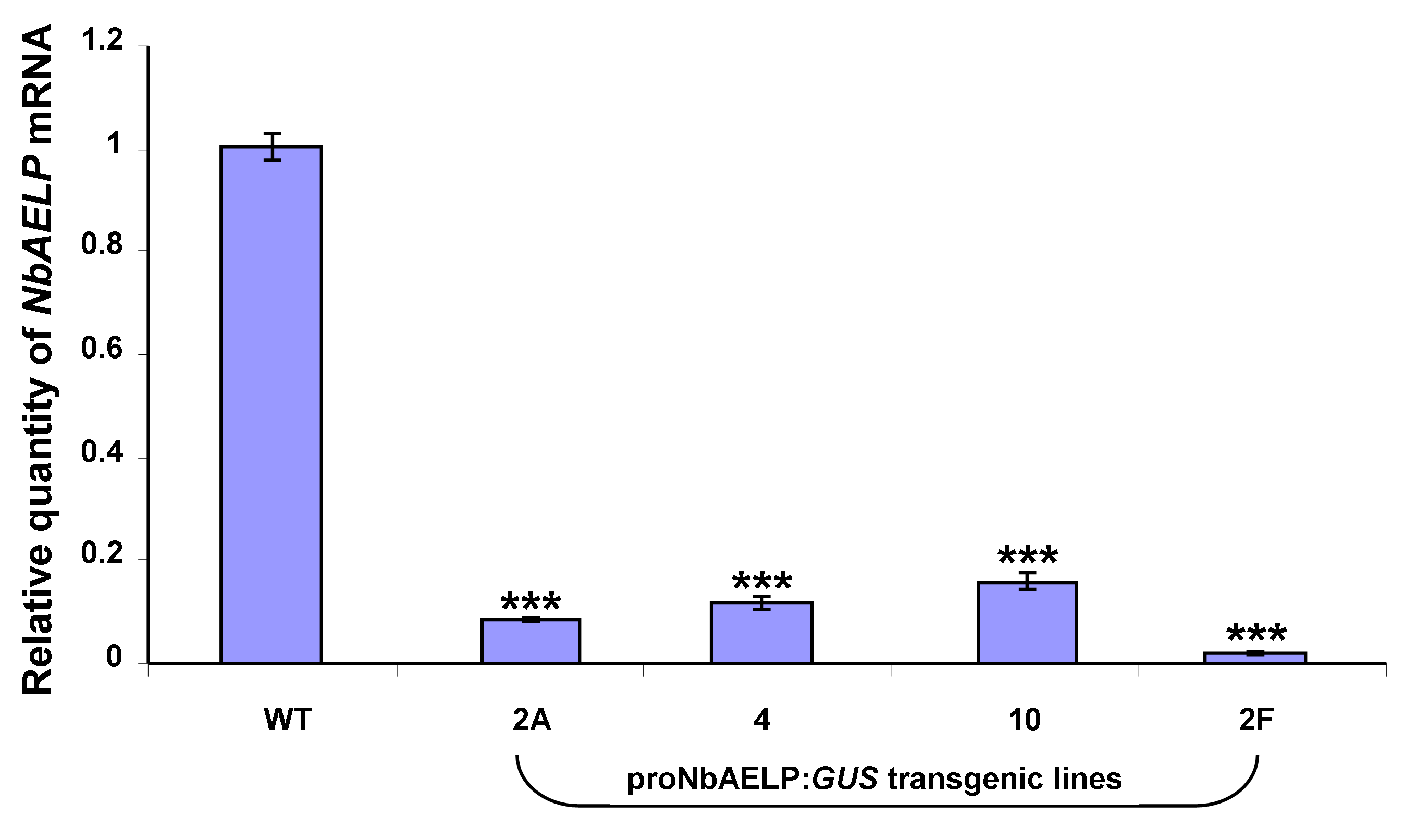

Supplement: Figure S6 — NbAELP and NbPME mRNA content in the leaves of proNbAELP-GUS N. benthamiana transgenic lines compared with WT control plants; qRT-PCR analysis of the mRNA levels in the leaves of proNbAELP:GUS transgenic lines 2A, 4, 10, 2F, and 11 of generation T1. [file Image6.TIF]

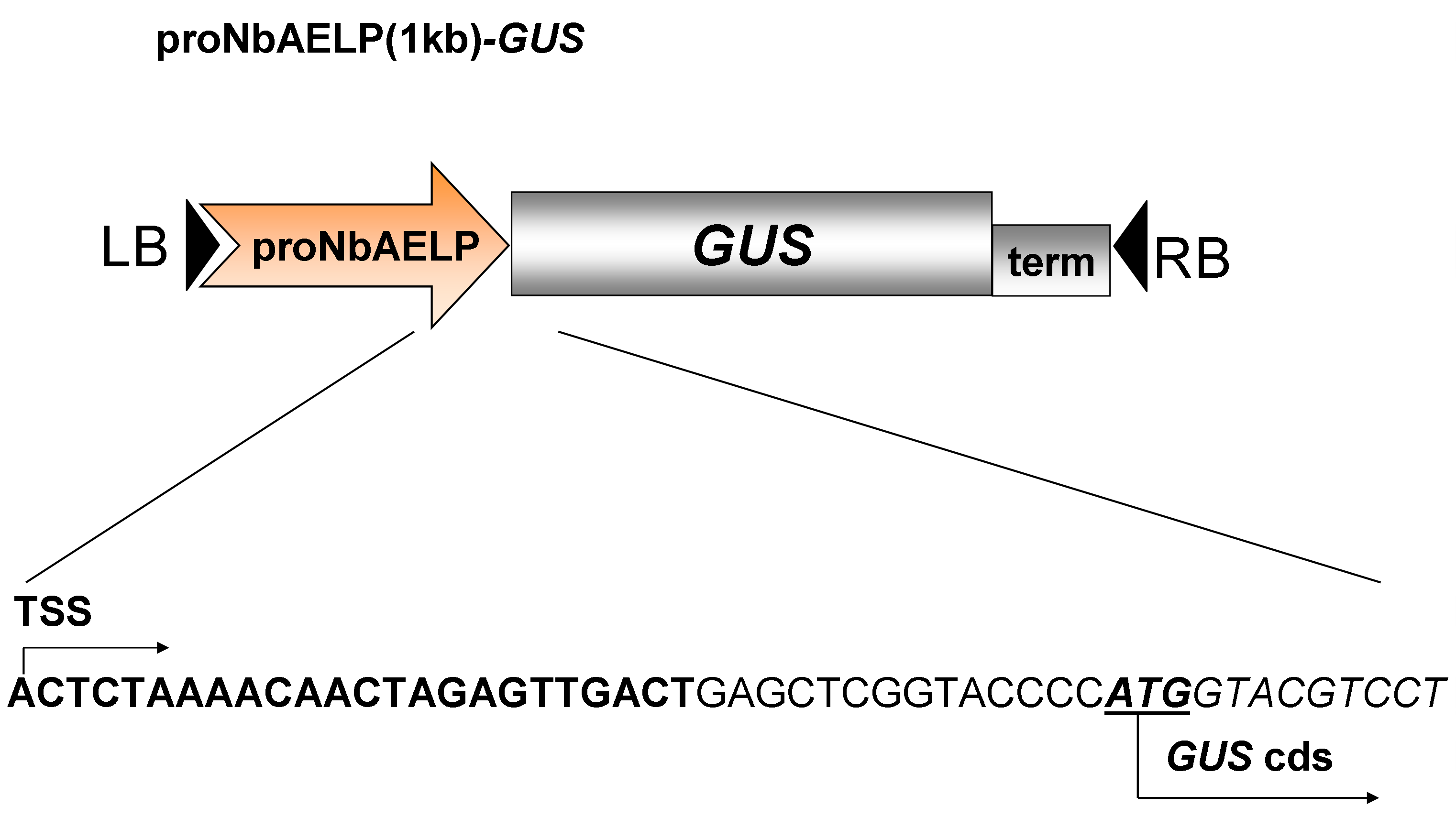

Supplement: Figure S7 — 5′RACE analysis of mRNA of proNbAELP:GUS transgenic plant. Schematic structure of the proNbAELP:GUS genomic insertion comprising the following elements: LB and RB, the left and right T-DNA borders, respectively; proNbAELP, 1-kb-proNbAELP; term, the 35S terminator of transcription. The transcription start site (TSS) and GUS coding sequence (cds) are indicated. [file Image7.TIF]

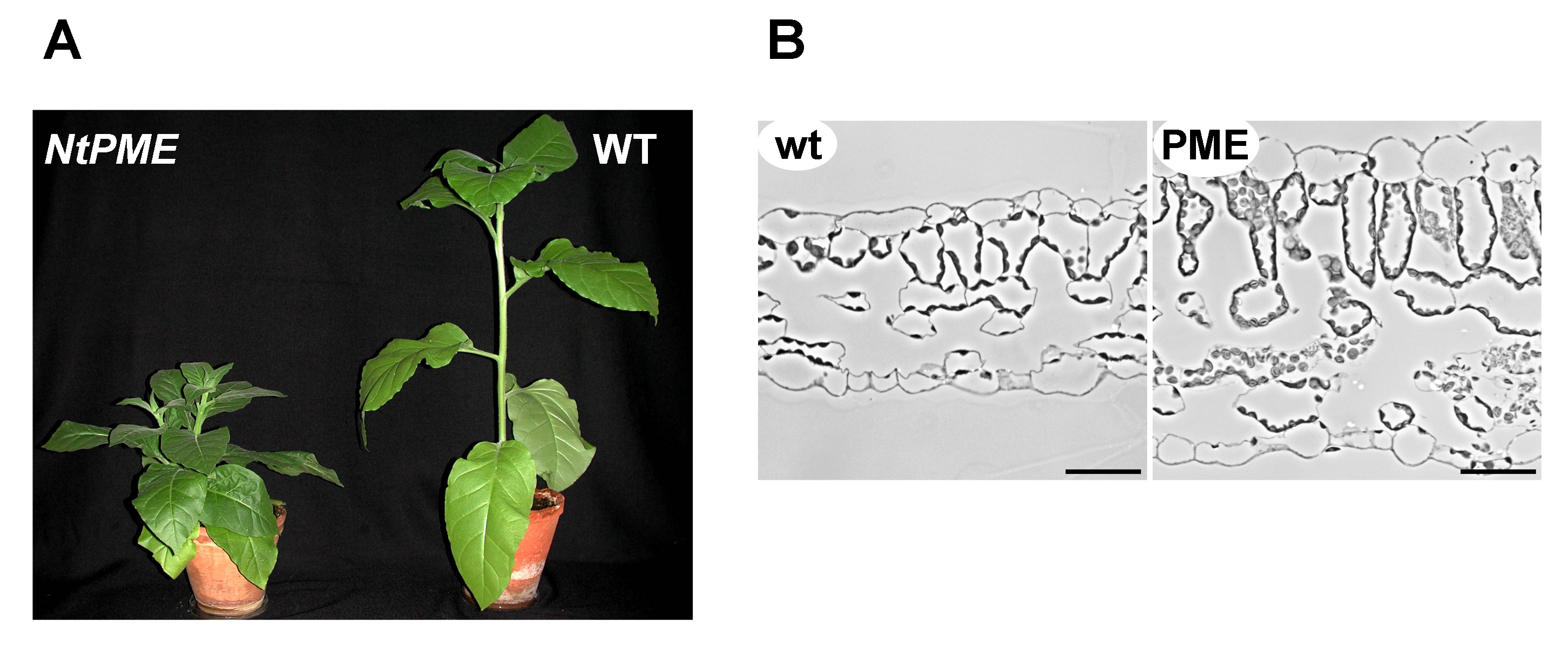

Supplement: Figure S8 — Increased PME enzymatic activity in the CW results in Nicotiana plant dwarfism. (A) Aerial phenotype of the 35S-NtPME transgenic tobacco at 15 weeks after sowing compared with WT control plants. (B) Transverse section of Epon-embedded leaf tissue from 35S-NtPME transgenic tobacco; bar = 50 μm. [file Image8.TIF]

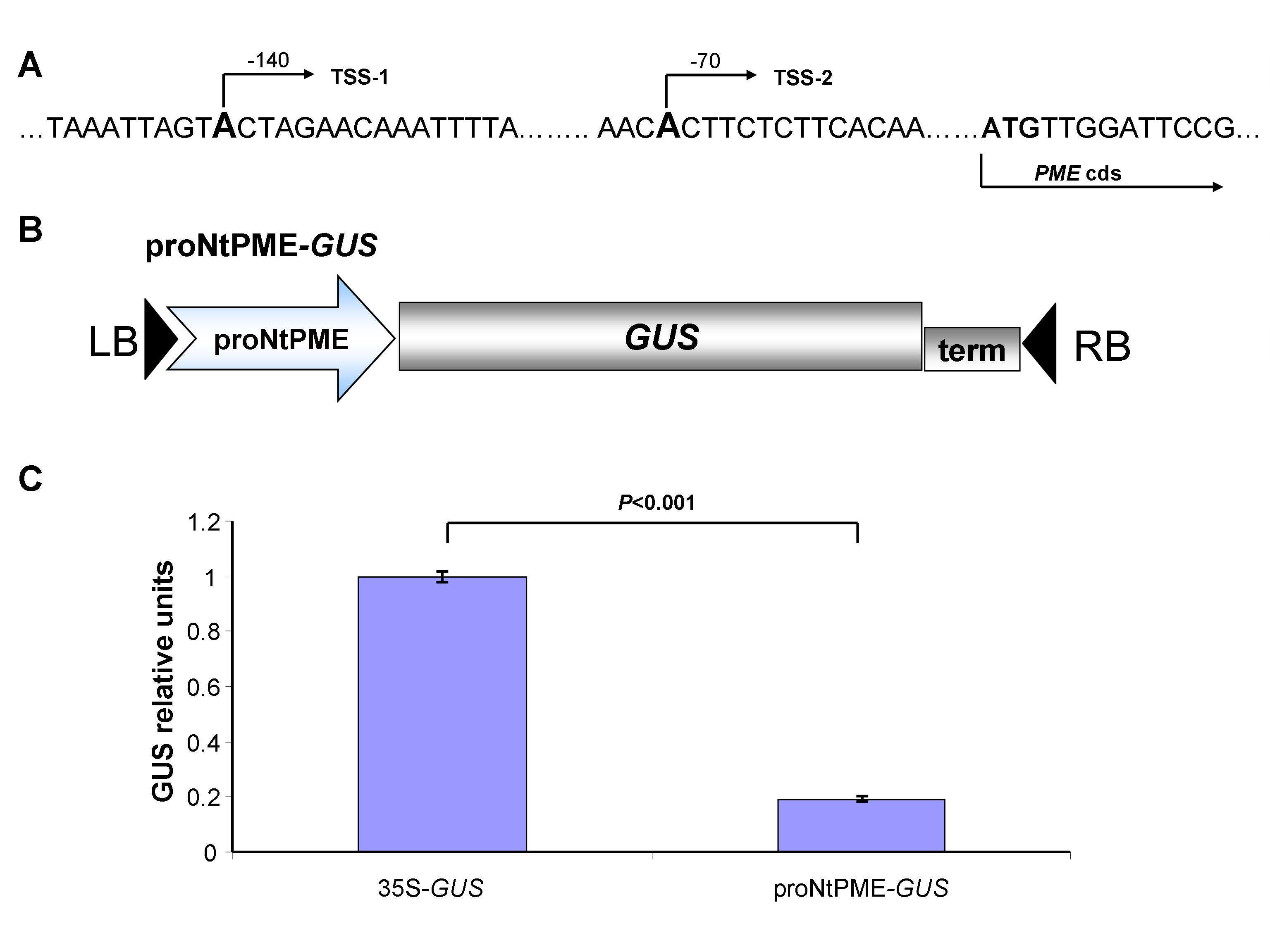

Supplement: Figure S9 — A 1.75-kb NtPME gene upstream sequence directs GUS mRNA synthesis. (A) Schematic representation of the upstream NtPME gene sequence (proNtPME) with two transcription start sites designated as TSS-1 and TSS-2. The start of PME coding sequence (cds) is indicated. (B) Schematic representation of the binary proNtPME-based GUS encoding vector proNtPME-GUS. (C) Comparison of the GUS activity in N. benthamiana leaves infiltrated with proNtPME-GUS or with 35S-GUS. The GUS activity, measured as relative light units, was normalized to the amount of plant total soluble protein. The mean values (with SE bars) for five independent experiments are shown. [file Image9.TIF]

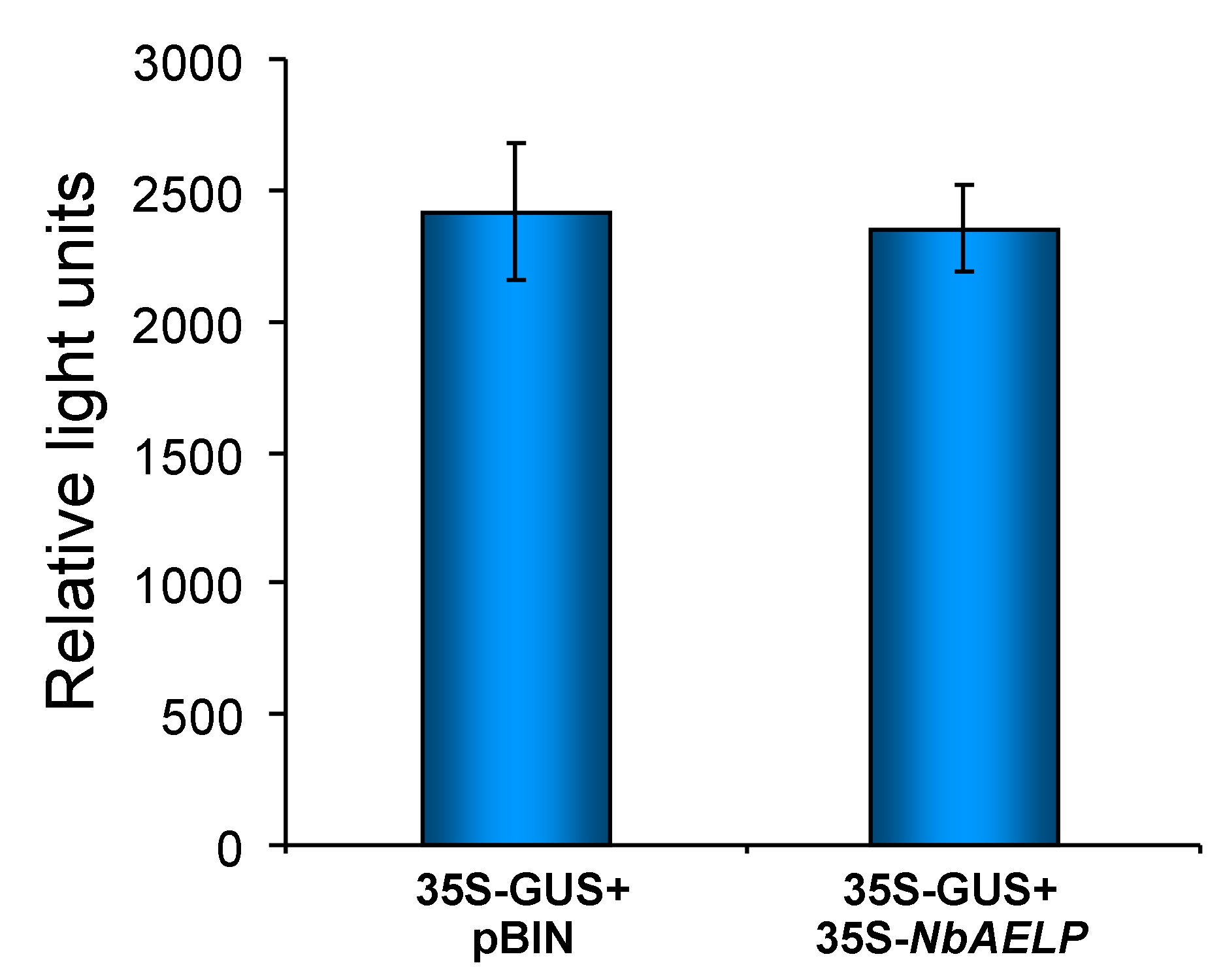

Supplement: Figure S10 — NtAELP has no effect on 35S-directed GUS synthesis. [file Image10.TIF]

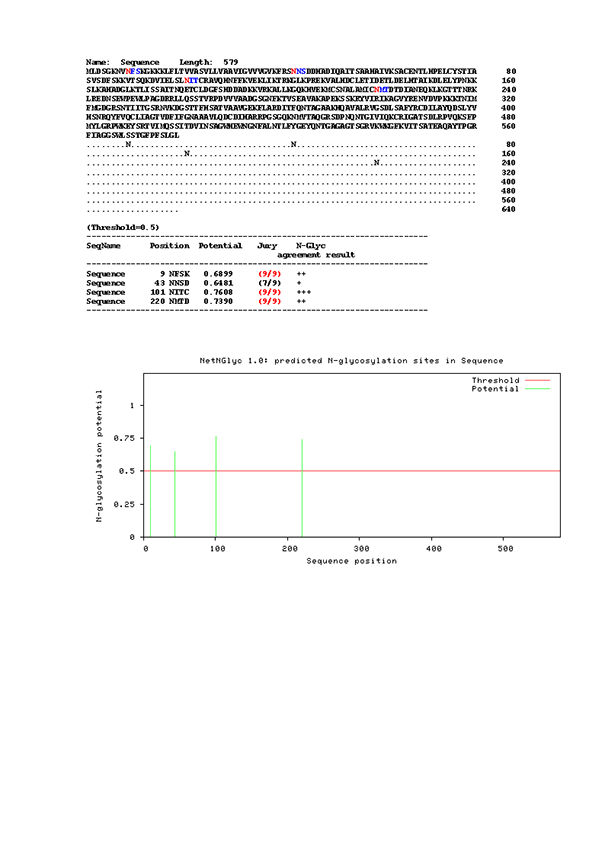

Supplement: Figure S11 — Predicted NbPME N-glycosylated sites with NetNGlyc 1.0 Server (http://www.cbs.dtu.dk/services/NetNGlyc/) are marked with bold red (Asparagines) and blue (Asn-Xaa-Ser/Thr sequons). [file Image11.TIF]
