# Supplementary material for: The Intergenic Interplay between Aldose 1-Epimerase-Like Protein and Pectin Methylesterase in Abiotic and Biotic Stress Control
Source: Front Plant Sci. 2017 Sep 25;8:1646. doi: 10.3389/fpls.2017.01646 (PMC5622589; doi:10.3389/fpls.2017.01646)
Supplement: Table S1 — Oligonucleotides used for cloning, 5'RACE and “genome walking.” [file Table1.DOC]

| Primer | Nucleotide sequence |
| --- | --- |
| FL_SalI_r | GTCGACATCCTTATAGTCTTTATCATCATCATCCTTATAGTCGCCTTTCTTG |
| FL2_SalI_r | GCTATTTATCATCATCATCCTTGTAGTCG |
| FL2_SalI_d | TCGACGACTACAAGGATGATGATGATAAATAGCTGCA |
| G05 prom Dir2 | ATAGAAGTTAATCTGAGCTCG |
| G05_ Rev6 | CTCTAACACTAGAAACAGCAC |
| G05_ Rev7 | ACCAATCTTTGAAGACATAGTC |
| G05_ Rev8 | TTTAGAGTGACTTAGTGGAGC |
| G05_ Rev10 | CGATTTTGTTGTTGAAGAAGGG |
| G05_ Rev11 | ATCTATAGAGTTGTTCAAGAATTG |
| G05_ Rev12 | CATCTTTTATGCCAAATTATGGG |
| GUS rev1 | CACTTTGCCGTAATGAGTGACC |
| GUS rev2 | CAACCTTTCGGTATAAAGACTTC |
| NbAELPpr (HindIII-) | AAGCTTTAACCTAGTTTATAAGA |
| NbAELPpr (SbfI+) | CCTGCAGGATAGAAGTTAATCTGAGCT |
| NbAELPpr(NheI+) | GCTAGCATAAACTAGGTTAAAG |
| NbAELPpr(SacI-) | GAGCTCAGTCAACTCTAGTTGTTTTAGAGTG |
| PME_rev1 | TATGGCCGTGTGGAGTTCGT |
| PME_rev2 | CGGCCCTGCAGGTGATATT |
| PME_rev3 | CTGCAGAGGTAATGGCCTGTAT |
| PME_rev4 | ACTACTCCGATAACTGCTGC |
| PME_rev5 | CTTTGGAAAAATTAACGTTC |
| PME_rev6 | TCCGGCGAAGAAATCTTTGA |
| PME_rev7 | TAATTTGGAGCAGCTAATTAG |
| PME_rev8 | CCTTTTTTAATTAATTTATCTC |
| PME_rev9 | TAATTTTAAAAGTCTTATTTAACA |
| PMEpr_D | TCCTCTTATCGTTTTATTTAA |
| PMEpr_HindIII+ | AAGCTTCCTCTTATCGTTTTATTTAA |
| PMEpr_NcoI- | CCATGGAAATTAACGTTCTTGCCGGAAT |

**Table S1. Oligonucleotides used for cloning, 5’RACE and “genome walking”**
