# Supplementary material for: The Intergenic Interplay between Aldose 1-Epimerase-Like Protein and Pectin Methylesterase in Abiotic and Biotic Stress Control
Source: Front Plant Sci. 2017 Sep 25;8:1646. doi: 10.3389/fpls.2017.01646 (PMC5622589; doi:10.3389/fpls.2017.01646)
Supplement: Table S2 — Oligonucleotides used for qPCR. [file Table2.doc]

**Table S2. Oligonucleotides used for qPCR**

| **Gene** | **Forward Primer** | **Reverse Primer** | **PCR Size, Bp** | **Acquisition temperature,^o^C** |
| --- | --- | --- | --- | --- |
| 18S rRNA | ACGGCTACCACATCCAAG | ACTCATTCCAATTACCAGACTC | 116 | 50 |
| GFP | GCAGAAGAACGGCATCAAG | GCTCAGGTAGTGGTTGTCG | 138 | 52 |
| GUS | CCTGCGTCAATGTAATGTTC | CGTATTCGGTGATGATAATCG | 192 | 53 |
| NbAELP | CTATGTCTTCAAAGATTAGTCTG | GGACGGAAATGATTGTGGC | 159 | 50 |
| PME | ATCCTTGGATTCCGGCAAGAACGT | AAACACTTGCAATTGTAGAGTAAC | 180 | 54 |
| TMV MP | ggtgtgagcgtgtgtctgg | gcgtcctgggtggttatagc | 137 | 54 |
| RCA | CATCTGTTGCCTCTTCATC | GCGTTCTCTTGCTTGTTG | 165 | 53 |
| AtpC | GCTCTTGGCATTGATTACAC | GTCAACCTCTTCACTCACG | 177 | 59 |
